# Nanotechnology-Assisted Cell Tracking

**DOI:** 10.3390/nano12091414

**Published:** 2022-04-20

**Authors:** Alessia Peserico, Chiara Di Berardino, Valentina Russo, Giulia Capacchietti, Oriana Di Giacinto, Angelo Canciello, Chiara Camerano Spelta Rapini, Barbara Barboni

**Affiliations:** Faculty of Bioscience and Technology for Food, Agriculture and Environment, University of Teramo, 64100 Teramo, Italy; cdiberardino@unite.it (C.D.B.); vrusso@unite.it (V.R.); gcapacchietti@unite.it (G.C.); odigiacinto@unite.it (O.D.G.); acanciello@unite.it (A.C.); chiara.cameranospelta@studenti.unite.it (C.C.S.R.); bbarboni@unite.it (B.B.)

**Keywords:** nanoparticles, cell tracking, cell labelling, cell uptake, stem cell homing

## Abstract

The usefulness of nanoparticles (NPs) in the diagnostic and/or therapeutic sector is derived from their aptitude for navigating intra- and extracellular barriers successfully and to be spatiotemporally targeted. In this context, the optimization of NP delivery platforms is technologically related to the exploitation of the mechanisms involved in the NP–cell interaction. This review provides a detailed overview of the available technologies focusing on cell–NP interaction/detection by describing their applications in the fields of cancer and regenerative medicine. Specifically, a literature survey has been performed to analyze the key nanocarrier-impacting elements, such as NP typology and functionalization, the ability to tune cell interaction mechanisms under in vitro and in vivo conditions by framing, and at the same time, the imaging devices supporting NP delivery assessment, and consideration of their specificity and sensitivity. Although the large amount of literature information on the designs and applications of cell membrane-coated NPs has reached the extent at which it could be considered a mature branch of nanomedicine ready to be translated to the clinic, the technology applied to the biomimetic functionalization strategy of the design of NPs for directing cell labelling and intracellular retention appears less advanced. These approaches, if properly scaled up, will present diverse biomedical applications and make a positive impact on human health.

## 1. Introduction

Cell tracking refers to the investigation of living cells over a period allowing the visual depiction, characterization, and quantification of biological processes at the cellular and subcellular levels within intact living organisms. A fundamental property of any real-world object is that it extends in both space and time. This is particularly true and applicable for living organisms in which the study of subjects in three dimensions and the possibility to track it over time (denoted as 3D or 4D) assumes great significance.

**Figure 1:**
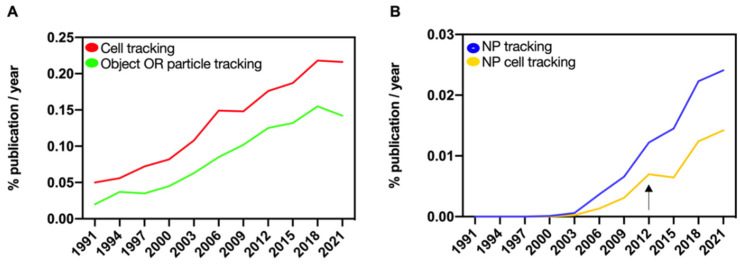
Scientific community interest in tracking related research. Percentage of publications in Web of Science (WoS) database as function of publication year for the indicated combinations of keywords with the field tag «Topic». “OR” was used as Boolean operator to combine the keywords “object” and “particle tracking” and to lunch the search in WoS database. The plot shows the exponentially increasing interest in cell, particle, or object tracking (**A**) as well as tracking with nano systems (**B**) in the biomedical and related literature. The impact of the intrinsic growth of the number of publications was weighed and corrected by plotting percentages.

Research community interest has grown exponentially in the last decades concerning the possibility of tracking objects (Figure 1A) [1,2,3,4,5,6] to automatically follow hundreds to thousands of cells. Imaging has provided the elective approach to enable cell tracking and it assumes the labeling of the target cell with objects, otherwise defined as contrast agents, with the aim of achieving contrast between the cells of interest and the other cells of the organism. Interestingly, nanoparticle (NP) systems have received great attention as the contrast agents to be used for cell labeling and followed by means of imaging techniques. Although the first attempts to automate the tracking of cells using NPs date back at least 20 years, the development of more advanced tracking methods really took off in the past decade (Figure 1B).

Two main components have a big impact in defining the approaches for cell tracking: the NP systems and the imaging technologies adopted to assist in cell tracking. NP systems are characterized by particles or constituents with unique features making them attractive for tracking purposes: (1) a small size (range diameter between 1 and 100 nm) and (2) a surface to mass ratio, functional to adsorb and efficiently carry compounds including peptides, fluorescent probes, and drugs that can be used as tracers, therapeutic tools or cell uptake enhancers [7].

Due to their nanoscale dimensions, NPs can be easily transported across cell membranes and reach the crucial subcellular organelles [8]. Furthermore, a high surface area-over-volume ratio enhances their interaction with cellular components [9]. In addition, NPs offer attractive features since their structure and chemical properties can be modified to facilitate cellular incorporation and because they can carry a high payload of the relevant labels into cells, thus generating high contrast signals to be followed by imaging.

Aside from NPs, the selection of appropriate imaging modalities assumes a crucial role in tuning the effective cell tracking approaches. Many advances have been achieved by developing new optical imaging probes, which have been useful for setting up multimodal imaging systems, and by adopting sophisticated optics and optical systems able to overcome the resolution limitations imposed by the scattering and absorption properties of tissue [10].

Overall, NP systems and imaging techniques can be considered as two closely related components of any cell tracking approach, which, if well managed, have an important impact in the improvement of current imaging resolution and sensitivity. The cell-tracking imaging platform choice relies on the type of contrast agent used for cell labeling and vice versa. Currently, the most suitable platform categories for NP-based imaging are magnetic resonance imaging (MRI), X-ray (also known as X-ray computed tomography and abbreviated as CT), optical imaging (endoscopy and fluorescence near-infrared or bioluminescence-based imaging methodologies), ultrasound, radionuclide molecular imaging and photoacoustic imaging (PAI), a hybrid technology combining optical and ultrasound imaging [11].

Specifically, MRI and CT are the two most used and clinically approved techniques for in vivo cell tracking, whereas the other imaging approaches have been primarily investigated in the last decade in translational research protocols showing a great potential for their clinical application. Cell tracking applications can be found in different areas of biomedicine ranging from the imaging of tissue replacement cell therapy (regenerative medicine) to cancer diagnosis and treatment (cancer medicine).

In regenerative medicine, cell tracking is a key technology for the development and optimization of cell therapy for the replacement or renewal of damaged or diseased tissue using transplanted cells [12]. In the cancer context, it helps early-stage cancer diagnosis and therapeutic imaging of tumor foci by improving accuracy and sensitivity of the current clinical protocols [13].

Several NPs with unique magnetic and/or optical properties have been investigated preclinically for real-time, in vivo cell monitoring [11]. These NPs include inorganic (with metal, ceramic, or semiconductor elements), organic (with polymeric or lipidic elements) and hybrid/composite (with both inorganic and organic elements) NPs. Of note, since the current imaging technology of choice for clinical translation is MRI, NPs with magnetic properties have been also tested for clinical application. Consistently, 11 studies employing NPs with magnetic properties for intra-operative procedure monitoring and/or diagnosis purposes are currently in clinical trial phases (Supplementary Materials Table S1). 

Different cell tracking approaches have been developed including the ability to track NPs enclosed in cell membranes, cell derivates (microvesicles) or actively and directly incorporated by the cells. The last, showing great potential for application in the field of cell tracking with imaging [14,15,16]. 

**Figure 2:**
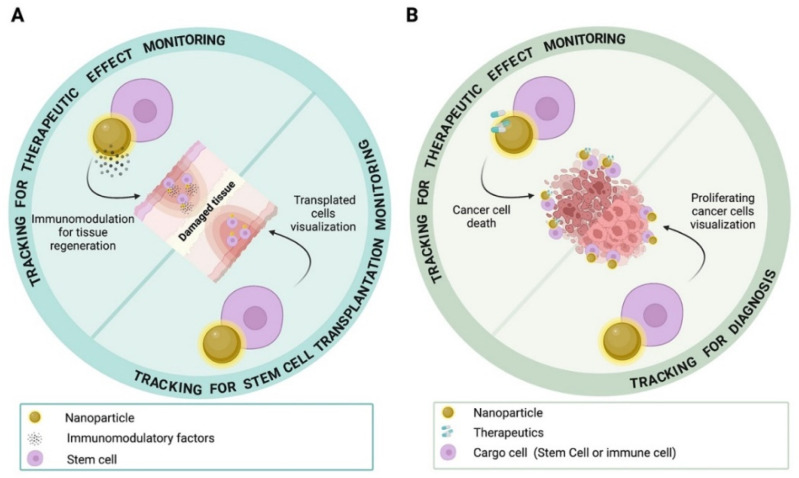
Direct cell tracking approaches suitable for imaging of cancer and cell transplantation for tissue regeneration, and related objectives. (**A**) Stem cells enclosing NPs can be used for monitoring transplantation events, as well as for directing therapeutic effects by releasing immunomodulatory factors into the tissue site requiring regeneration. (**B**) Stem cells or immune cells enclosing NPs can be adopted for homing in on tumors by allowing diagnosis and/or therapy. Figure created with BioRender. Available online: https://biorender.com/ (accessed on 25 March 2022).

In the field of regenerative medicine, the tracking of stem cell transplantation procedures and regenerative performance through imaging have been investigated with success by employing direct cell labeling methods based on the introduction of the NPs working as a contrast agent into the cells to be transplanted prior to in vivo injection [17]. Stem cell tracking approaches enhancing the regenerative properties of stem cells to be transplanted have also been developed by exploiting the possibility of incorporating into these cells NPs bearing specific moieties with an immunomodulatory role [12] (Figure 2A). 

Of note, this strategy emerged as also applicable in the field of cancer imaging, where the native attitude of stem cells to sense and home in on tumor foci as well as the ability of some immune cells (e.g., macrophages or T cells) to be recruited by tumor-derived inflammatory stimuli, were exploited by using these cells as vehicles for NPs and thus, as a tracer guiding the identification of cancerous districts [18,19,20,21]. Moreover, because of the availability of specific NP systems enclosing therapeutics within their structure, this approach was also found to be suitable for monitoring the therapeutic effects elicited by the NPs conveyed by the cargo cell or directly incorporated by the cancer cells to be targeted [22] (Figure 2B). 

The application of cell tracking approaches has a big impact on the improvement of currently available diagnostic and therapeutic imaging protocols. In the context of regenerative medicine, NP-based cell tracking through imaging provides a safe way to monitor the viability, proliferation and differentiation of transplanted cells by enabling the guidance of cell replacement and testing the therapeutic success of the adopted transplantation procedure. 

With respect to cancer medicine, NP-based cell tracking through imaging represents an invaluable tool to provide a rapid and sensitive detection of cancer foci, thus acquiring a relevant role in early tumor diagnosis [13,23], consequently yielding greater chances for cancer treatment, and extending life expectancy. Furthermore, it can be exploited to monitor the response to specific theranostics, allowing the assessment of disease state [24]. Interestingly, some recent evidence has shown a possible application in image-guided surgery, enabling the intra-operative detection of any residual tumor mass to be removed and/or therapeutically targeted [25,26,27]. Despite their great potential, there are currently some limitations regarding their application for in vivo cell tracking at the translational level yet to be overcome [28]. 

This review aims to provide an overview of the nanotechnologies and methodologies eligible for cell tracking which have been investigated in the last decade, the window period identified as critical for the development of NP-based tracking strategies, with emphasis on (1) the specific NP characteristics responsible for effects on cell labeling, consequently defining the effectiveness of cell tracking, and (2) the approaches and mechanisms of NP incorporation into cells.

Consequently, the next paragraphs will focus on the following key elements: the types of NPs currently adopted in cell imaging (Section 2); the NP functionalization strategies most used to improve cell targeting, cell uptake, therapeutic tracking, and imaging performance (Section 3); the approaches suitable for in vitro, in vivo and ex vivo tracking of NP delivery (Section 4); the uptake mechanisms of the NPs (Section 5) and the cellular systems mostly described for NP loading (Section 6).

All the above key aspects have been inferred from research evidence collected with a systematic literature review performed in Web of Science (WoS) database during the last decade which focused on the investigation of NP-based cell tracking strategies.

## 2. NP-Based Cell Tracking Derived Bibliographic Database 

Literature data published in peer-reviewed international papers indexed in the advanced search of WoS [v.5.35] “Core collection” archive (https://apps.webofknowledge.com/WOS_AdvancedSearch (accessed on 3 July 2021) in the last 10 years were considered.

Specifically, three lists were created by searching for the following key words and filtering for original research articles:

1. Nanoparticles, cell tracking methods, fluorescent dyes;
2. Nanoparticles, cellular uptake, cell labelling, staining;
3. Tomography, magnetic resonance, nanoparticles, stem cell homing.

The words “AND” and “OR” were used as Boolean operators, “TS” as field tag. 

The lists of papers originating from each key word search were then matched to create the final database of citations. 

Overall, the adopted bibliographic approach allowed us to collect 151 original studies focusing on the investigation of strategies for direct cell labelling and tracking with NPs (Supplementary Materials Table S2). Ninety-eight of the total citations (64.5%) characterized the approaches to incorporate NPs into vehicle cells to be used to sense target cells or body districts after in vivo delivery into the body. In these studies, the aim was to find the most suitable way to improve uptake efficiency without affecting cell cargo wellness. 

Fifty-four of the total citations (35.5%) investigated the effect of NP incorporation into cell models to be therapeutically targeted. In these studies, the aim was to find a protocol that could be effective in targeting desired cells, monitoring NP uptake and the cytotoxic effect of NP cell internalization (see Supplementary Materials Table S2). 

Furthermore, based on the adopted cell models, the collected studies could find application in the field of cancer and tissue regeneration imaging. Specifically, 72 of the total citations were applied to cancer imaging, 57of the total citations to tissue regeneration imaging, whereas 23 of the total citations adopted models suitable in both fields (see Supplementary Materials Table S2).

## 3. NP Features and Cell Tracking Applications for Tissue Regeneration and Cancer Imaging

**Figure 3:**
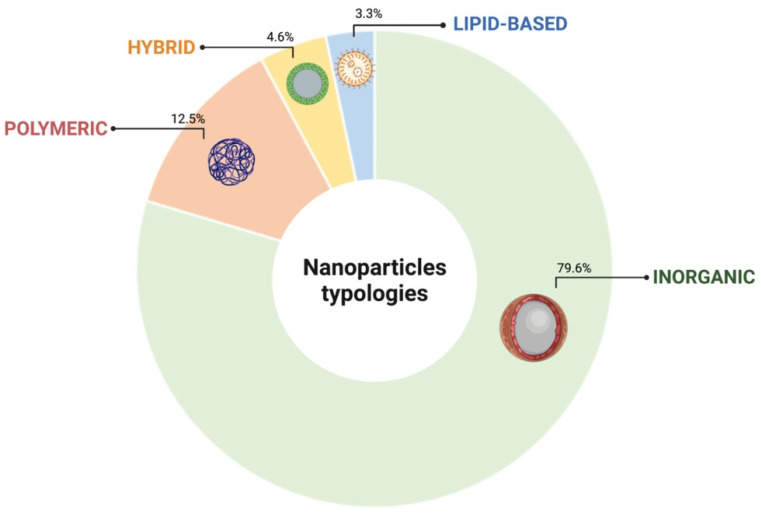
Classification of NPs based on the constitutive element. Classes and subclasses of nanoparticles based on their chemical properties (inorganic, polymeric, hybrid and lipid-based). Percentage (%) refers to the typology of NPs with respect to the total number of NPs identified in the literature survey. Figure created with BioRender. Available online: https://biorender.com/ (accessed on 25 March 2022).

The literature survey allowed us to identify four distinctive classes of NPs as useful for developing suitable cell tracking platforms: inorganic, polymeric, hybrid and lipid based. The inorganic category resulted in the most represented NP typology in this survey, accounting for the 79.6% of the total collected citations, followed by polymeric (12.5%), hybrid (4.6%) and lipid based (3.3%), respectively (Figure 3). The prominent representation of the inorganic typology reflects the fact that these NPs are the ones best suited to cell tracking through the most characterized imaging platforms to date. 

### 3.1. Inorganic

Inorganic NPs are characterized by a typical core/shell structure which can assume different shapes: spherical, rods, wires, tubes, pyramids, and stars. The core size of these nanoparticles is approximately 3–6 nm and a coated system (shell) increases its size to 20–150 nm. The core can contain metals, other chemical elements or fluorescent dyes encapsulated in silica. The core defines the magnetic, electronic, fluorescence and optical properties of the NP [28]. The shell is usually made of metals or organic polymers that (I) protect the core from chemical interactions with the external environment, (II) serves as a substrate for conjugation with biomolecules (antibodies, peptides or oligonucleotides) and (III) preserves NP stability avoiding aggregation [29,30]. Due to their magnetic, radioactive, X-ray absorption or plasmonic properties, inorganic NPs are used for diagnostics and imaging purposes and most of them display good stability [31] and biocompatibility [32]. 

Four main subcategories of inorganic NPs applicable for cell tracking were identified by this survey, based on the nature of the core element:

(I) Metal-based NPs, accounting for 31.5% of the collected inorganic NPs, include silica, manganese, gold, silver, lanthanide, molybdenum, ruthenium, rubidium, gadolinium, and zinc elements.
(II) Metal oxide-based NPs, representing 64.5% of the collected inorganic NPs, enclose iron oxide, superparamagnetic iron oxide (SPIO), ultrasmall superparamagnetic iron oxide (USPIO), titanium oxide and cobalt iron oxide elements.
(III) Metal sulfide or phosphide-based NPs, accounting for 2.4% of the collected inorganic NPs, comprise quantum dots.
(IV) Mineral-based NPs, accounting for 2.4% of the collected inorganic NPs, take in hydroxyapatite and selenium elements.

### 3.2. Polymeric

Polymeric NPs are particles within the size range from 1 to 1000 nm and are constituted by a polymeric matrix core which can be loaded with bioactive molecules. They are categorized into two forms, spheres (the bioactive molecule is dispersed within a polymer matrix) and capsules (the bioactive compound is placed in the core of the particle covered by a layer of polymer). Polysaccharides and proteins are the commonly used materials for the fabrication of polymeric nanoparticles. The polymer provides biocompatibility and protection to the active molecules to the delivery site [33].

The most used polymers for the constitution of polymeric NPs collected by this survey were poly(ethylene glycol) (PEG; 33% of the collected polymeric NP), poly(lactide-co-glycolide) (PLGA; 24%) and polystyrene (PS; 13%). Although to a lesser extent, other polymers were also identified: poly(epsilon-caprolactone) (PCL), poly(lactide) (PLA), poly(2-methacryloyloxy) ethyl phosphorylcholine (PMPC), poly(glycerol-co-sebacate) (PGS), poly(diphenylamine)-poly(ethylene oxide) (PDPA-PEO) and poly[2-methoxy-5-(2-ethylhexyloxy)-1,4-phenylenevinylene] (MEH-PPV). Of note, these polymers have been used alone or in combination to design polymeric NPs for cell tracking.

### 3.3. Hybrid

Hybrid NPs are constructed from at least two different NPs, to overcome the limits of single-component nanoparticles, to improve properties, to achieve new properties not possible for single nanoparticles, and/or to achieve multiple functionalities for single nanoparticles. Size range can be variable depending on the NP typologies used for their fabrication [34].

### 3.4. Lipid-Based

Lipid-based NPs, defined as colloidal carriers for bioactive molecules, are assembled to constitute different structures: liposomes with size < 200 nm, solid lipid NP (SLN, solid lipids), and nanostructured lipid carriers (NLC, a combination of liquid and solid lipids) with sizes ranging from 10–1000 nm [35,36,37,38].

Liposomes are spherical vesicles with an aqueous core and bilayer lipid membrane. They have the capacity to encapsulate diverse bioactive compounds, which can be included into the aqueous core or at the bilayer interface [39].

SLNs are generally spherical in shape and consist of a solid lipid core stabilized by a surfactant. This construct can be used to deliver both hydrophilic and hydrophobic bioactive molecules [40].

Mixing solid lipids with small amounts of liquid lipids allows structural rearrangements of the matrix-generating NLC formulations to be produced, improving their properties in this respect, while maintaining the original benefits of SLNs. Of note, recently, cell-derived membrane lipidic vesicles with a small size range (40–100 nm) have been considered as alternative naturally derived lipid NPs versus the synthetic lipidic formulation [16].

With respect to the application of the different NP typologies in the field of cancer imaging or tissue regeneration imaging, the literature survey revealed that polymeric and lipid-based NPs are the most represented in the studies, pointing to the development of cancer imaging and therapeutic protocols; the hybrid category was applied for tissue regeneration imaging whereas use of the inorganic category was well documented for setting up both cancer and regenerative imaging protocols (see Supplementary Materials Table S1). Importantly, some of the collected studies, which will be further discussed below, moved a step forward in the characterization of the best NP platform for cell tracking by providing the adoption of in vitro and/or in vivo imaging procedures to be applicable in cancer or regenerative imaging.

### 3.5. NP-Based Cell Tracking for Imaging of Tissue Regeneration

In the context of tissue replacement cell-therapy imaging, protocols assuring the long-term monitoring of transplanted cells without affecting cell viability and/or properly modulating their state of differentiation depending on the tissue/organ to be targeted, have yet to be identified. Indeed, at present, clinical cell-tracking trials have only provided information on immediate cell delivery and short-term cell retention [41]. Overall, many key issues concerning labeling solutions able to augment the possibility of tracking cells enclosing NPs, avoiding label dilution during cell proliferation and improving the uptake and retention of NPs, as well as preserving the proper functions of the transplanted cell and their correct homing to the target site, remain to be addressed [42].

### 3.6. Labeling Solution Improving Cell Imaging

Over the last decade, direct cell labeling with inorganic NPs engineered with multiple tracers has received increased attention for improving the tracking and imaging performance of tissue and/or organ regeneration [39]. Indeed, the use of a combination of two or more chemical elements to be traced by means of different imaging techniques, known as the multi-labeling approach, represents a way to overcome the imaging detection limits of a specific technique. As some examples, inorganic gold NPs with red-emitting firefly luciferase (RfLuc)-based bioluminescence (BL) tags [43] and inorganic NPs with gold and gadolinium elements [44] have been developed for the noninvasive labeling and tracking of human mesenchymal stem cells (hMSCs) in a mouse model of pulmonary disease. Specifically, multimodal imaging with CT, MRI and BL was successfully adopted to characterize the in vivo migration, homing, functions, and survival of transplanted hMSCs, giving further insight for the treatment of pulmonary fibrosis.

Inorganic iron oxide NPs modified with melanin and Fe ions were successfully used for dual modal imaging through MRI and photoacoustic (PA) methods of engrafted bone marrow-derived mesenchymal stem cells in mouse models [45]. Multilayered inorganic NPs constituted by a combination of mesoporous silica, iron oxide and gold elements were successfully used to multilabel umbilical cord mesenchymal stem cells in ischemic mouse brain models [46]. Moreover, inorganic iron oxide labeled with a fluorophore have been used to trace in utero hematopoietic cell transplantation in a canine animal model, offering a potential approach for early intervention for the treatment of diseases before birth [47].

Interestingly, Geburek and colleagues experimented on an additional way for multilabeling by using adipose-derived mesenchymal stem cells traduced with a reporter gene for GFP and co-labelled with iron particles for tracking after the intralesional treatment of artificial equine tendon lesions [48]. A similar approach was used to efficiently track the homing of magnetically labeled adipose-derived stem cells [49] and human umbilical cord-derived mesenchymal stem cells [50] expressing GFP into mouse carotid artery and cutaneous injury sites, respectively. In contrast, Wu and colleagues suggested combining the reporter gene directly with the NPs. In this case, the adoption of inorganic iron oxide NPs complexed with polymers acting as a cargo for the luciferase system and or a fluorescence protein as reporter genes was shown to be effective for an MRI-visible gene delivery agent which could effectively label MSCs, providing the basis for bimodal bioluminescence and MRI tracking of transplantation to solve acute liver injury [51].

Hybrid NPs have also been adopted for generating a platform for multimodal imaging. Fluorinated polymeric NPs combined with fluorine as the MRI contrast agent and functionalized with a fluorophore (N-fluoresceinyl) maleimide) as optical contrast moiety were used for tracking MCS in vivo [52].

Advances in multimodal labeling and tracking of cell transplantations have also been made by functionalization of polymeric NPs. Zhang et al. identified how the fluorescent imaging of human mesenchymal stem cells that have endocytosed fluorescent polymeric NPs can be directly and clearly captured in the one-photon and two-photon modes, offering the possibility of the direct monitoring of stem cells with high resolution and quantifying NP uptake, encouraging future quantitative clinical assessment in imaging-guided cell therapies [53]. Similarly, the exploitation of the one-photon and two-photon imaging properties of polymeric NPs loaded with red phosphorescence dye of bis(2-methyldibenzo[f,h]quinoxaline) (acetylacetonate) iridium(III) (Ir(MDQ)2acac) provided a reliable approach for the efficient and sensitive tracking and monitoring of transplanted neural stem cells (NSCs) [54]. Multimodal tracking was also achieved by functionalizing NPs with fluorine, which served as contrast agent for MRI and as an effective platform to properly direct the transplanted MSC fate in a non-obese diabetic/severe combined immunodeficient mouse model [52], as well as for the tracking of immune and stem cells to be adopted for cellular therapies [55].

### 3.7. Labeling Solution Improving Cell Uptake and Homing

The labeling solutions aimed at improving cell uptake and homing to the desired site of the body have been investigated by functionalizing NPs with different biomaterials. Accordingly, the literature survey identified inorganic iron oxide NPs functionalized with several moieties including poly-lysine, dextran, zinc, protamine sulfate, heparin or poly-(l-lactide) as good platforms for direct label, home and track MSCs in preclinical models of wound healing [56], traumatic brain injury [57,58,59,60,61], after renal transplantation in mice [62], hepatic cirrhosis [63] and photothrombotic cerebral infarction [64] with MRI. Poly (dopamine)-coated iron oxide NPs were also used for noninvasive labeling, tracking, and targeted delivery of adipose tissue-derived stem cells in mouse models of liver diseases [65].

Efficient strategies for the traceable homing of therapeutic NPs were developed by using hybrid NPs composed of glucocorticoid betamethasone phosphate (BMP) and the fluorescent dye DY-647 (BMP-IOH-NPs). Uptake of these NPs into macrophages was proved to efficiently drive treatment of inflammation with simultaneous in vivo monitoring of NP delivery [66].

### 3.8. Labeling Solutions for Long-Term Imaging and Modulation of Stem Cell Differentiation State

Protocols assuring long-term monitoring and targeted modulation of transplanted stem cell differentiation have been investigated by functionalizing NPs with moieties such as bioactive factors and/or nucleic acids able to manage cell fate upon their in situ release. Consistently, the literature survey identifies studies in which inorganic mesoporous silica NPs functionalized with dexamethasone [67] or a specific miRNA (miRNA-26a-5p) [68] (p. 2) resulted in effective tracking of bone marrow stem-cell homing and enhancing osteogenic differentiation in preclinical mouse models. Furthermore, polymeric NPs bearing the stromal factor-1α (SDF-1α) as the bioactive molecule known to regulate MSC homing and localization and the antimir138, with a key role in the promotion of osteogenic differentiation proved to be efficient for driving cranial bone regeneration by means of MSCs in preclinical mouse models [69].

Other studies have noticed a strong influence of intrinsic physicochemical properties of some types of NPs in directing stem cell fate by modulating the differentiation and proliferation processes. Accordingly, inorganic gold NPs modified with 11-mercaptoundecanoic acid as well as inorganic upconverting NPs have shown the ability to modulate lineage differentiation of rat bone marrow MSCs [70,71].

### 3.9. NP-Based Cell Tracking for Imaging of Cancer

A NP-based cell tracking system represents a critical element, which if well managed, might allow the improvement of the resolution and sensitivity of current imaging techniques applied for targeted cancer diagnosis and/or therapy. Currently, the most widely used diagnostic imaging tools for the clinical detection of cancer, such as X-ray, magnetic resonance imaging (MRI), computed tomography (CT), endoscopy, and ultrasound, can only detect cancer when there is a visible change to the tissue. By that time, thousands of cancer cells may have proliferated and even metastasized. In addition, current imaging methods cannot distinguish benign lesions from malignant lesions [72]. Furthermore, cytology and histopathology examinations are usually adopted to characterize malignant cells and/or tissues primarily identified through imaging techniques such as CT or MRI and cannot be effectively and independently applied to detect cancer at an early stage [73].

The exploitation of the different physicochemical properties of NPs and the possibility of functionalizing NPs with a plethora of bioactive molecules have been demonstrated to be crucial in innovating the procedures for cancer imaging and to allow targeted therapeutic NP uptake.

### 3.10. Labeling Strategies to Improve Targeted Cell Uptake and Tracking

Improved targeted cancer cell tracking has been achieved by functionalizing several types of NPs with moieties which enhance tracking resolution.

In this context, inorganic silica NPs conjugated with innovative fluorescent nanomaterials bearing excellent photostability and with specific miRNAs and/or active molecules with a high affinity for cancer cells were successfully adopted to trace cancer foci. In detail, the synthesis of fluorescent dye-doped silica NPs carrying miRNA-21 or PEG peptide were demonstrated to be effective in NP delivery and imaging in breast [74] and liver [75] cancer cells. miRNA21 was found to be one of the most represented in breast cancer tumors [76], whereas PEG has been found to effectively recognize tumor-marker carcinoembryonic antigens (CEAs) [77]. Furthermore, inorganic iron oxide NPs carrying therapeutic siRNAs were efficiently used for tracking siRNA-based gene therapy targeting the BCL2 and BIRC5 of oral cancer and glioblastoma cells [78].

Another class of NPs, polymeric NPs conjugated with liposoluble fluorescent probes (fluorescein, coumarin 6 or DiR probes) as contrast agents and with oleanolic acid [79] or emodin and heparin sodium [80] as targeting moieties, were also used to track liver cancer cells to be therapeutically targeted. The multifunctionalization of polymeric NPs with coumarin 6 as labeling moieties and with either a therapeutic moiety and a peptide (peptide-22) with a special affinity for the low-density lipoprotein receptor (LDLR) was also successfully used to facilitate drug penetration of the blood–brain barrier and to improve the visualization of targeted cellular uptake, avoiding a non-selective brain drug accumulation in a preclinical mouse model of glioblastoma [81].

Furthermore, the tracking of polymeric NPs labeled with a near-infrared dye (NIR) and conjugated with either a chemotherapeutic drug paclitaxel (PTX) and transferrin as the targeting moieties allowed the biodistribution of tumor cells incorporating NPs and the antitumor effect to be studied in a preclinical mouse model of cancer overexpression in the transferrin receptor [82]. Conjugation of polymeric NPs with NIR fluorophore dye has also been proved efficient in mediating the long-term tracking of cancer cells in vivo [83].

Strategies for cancer imaging have also been developed by adopting other types of NPs. Hybrid NPs constituted by glyceryl monooleate-coated magnetic nanoparticles (MNPs) combined with glyceryl monooleate were found to act as a better labeling and efficient tracking agent without affecting the inherent properties of the MSCs to be used for tumor homing in preclinical rat models [84].

Lipid-based micelle NPs carrying a fluorescent-tagged antibody targeting the breast cancer cell antigen HER2 showed highly efficient internalization into the target cell, good tracking performance and a cytotoxic effect [85].

### 3.11. Labeling Strategies to Improve Imaging Sensitivity

Methods to augment the possibility of localizing the homing of stem cells or other cell types bearing NPs to tumors are being investigated with the goal of enhancing tracking and treatment efficacy.

As some examples, cationic magneto-liposomes were used to magnetically label human blood outgrowth endothelial cells (BOECs) and follow their homing to map cancer foci by magnetic resonance imaging (MRI) [86]. Importantly, these cells have shown extensive promise as gene-delivery vehicles for cancer cell treatment.

Furthermore, the two- and three-photon luminescence capabilities and strong optical absorption of an innovative class of gold NP structures, known as gold nanocages, enabled the quantitative tracking of labeled hMSCs using two imaging procedures: two-photon microscopy and photoacoustic microscopy. Importantly, this bi-modal tracking enabled the locating of cells that had migrated to glioblastoma regions formed by subcutaneous injection in nude mice in vivo [87].

In addition, luciferase-expressing human adipocyte-derived stem cells (ADSCs) were also used as a vehicle for Indium-111 radiolabeled inorganic iron oxide NPs to produce cells with tri-modal imaging capabilities. The ADSCs’ homing and fate was monitored by using mouse models of breast cancer with bioluminescence imaging (BLI) as a measure of cell viability, magnetic resonance imaging (MRI) for cell localization and single photon emission computer tomography (SPECT) for cell quantification [88].

Dual-modal imaging has been investigated for improving the sensitivity of detection and accurate evaluation of benign and malignant lymph nodes by adopting hybrid NPs comprising superparamagnetic polymeric micelles conjugated with a fluorescent dye. These NPs showed efficient targeting and uptake by lymph node macrophages [89].

## 4. NP Functionalization Strategies for Cell Targeting, Cell Uptake, Therapeutic and Imaging Tracking

### 4.1. Key Factors for an Efficient and Targeted NP Uptake into Cells

Accurate cell tracking occurs if the NPs are correctly taken up by the cell to be targeted. The targetability and efficiency of the uptake are governed by the interactions between the NP surface groups and the plasma membrane antigens and/or receptors, which in turn depend on the density of the ligands and the antigens/receptors present on an NP and a cell, respectively [90]. NP functionalization with specific bioactive molecules (hereinafter referred as moieties) have been investigated to that purpose and those collected with the bibliographic survey were summarized in Table 1. Specifically, these moieties can elicit a targeted NP uptake by capturing specific cell biomarkers such as antigens/receptors (moieties with active action) or by enhancing NP permeation and retention (moieties with passive action) (Table 1).

### 4.2. Targeting and Uptake Moieties

Antibodies [91,92,93,94] with high specificity and affinity towards cancer cell surface molecules have been identified as active targeting and uptake moieties. Likewise, folic acid and riboflavin vitamins [95,96,97,98,99,100] have represented an attractive strategy for efficient NP uptake by cancer cells to be targeted, due to both overexpression of the folate and riboflavin receptors on the cancer cells and the rapid internalization of the receptor by receptor-mediated endocytosis [101,102].

Aptamers, consisting of small molecule DNA or RNA fragments that fold into well-defined 3D structures, have also been found to recognize specific receptors on the cell surface and mediate NP internalization [43,74,81,85,103,104,105,106,107]. The same is true for carbohydrates such as dextran and carbodextran [47,48,57,64,108,109,110,111,112,113,114,115,116], chitosan [69,117], glucose [118,119], beta cyclodextrin [120], and the glycoprotein transferrin [82] that have been mostly used for NP coating as a strategy to avoid immune response and, furthermore, to introduce active-targeting capabilities as well as increase cellular uptake [121].

Passive targeting and NP uptake have been efficiently achieved by polymers [56,60] and heparin [55,62] which, based on their biocompatibility, allow for enhanced NP permeation and retention.

Other types of moieties with passive action were quaternary ammonium cations [87], the polypeptide polylysine [49,58,63,64,122,123,124,125,126], and amino acids such as histidine [127,128], which, thanks to their positive charge, stabilize NPs and mediate the electrostatic interaction with the cell membrane, improving the endocytosis mechanisms. Similarly, some derivatives of vitamins, as an example TPGS compound [80], have been found to improve NP uptake by increase membrane permeability [129].

**Table 1:** NP surface targeting and uptake moieties.

Targeting and Uptake Moieties	Active or Passive Action	References
Antibodies, peptides	Active	[91,92,93,94]
Aminoacids	Passive	[127,128]
Aptamers	Active	[43,74,81,85,103,104,105,106,107]
Carbohydrates and glycoproteins (chitosan, beta cyclodextrin, dextran, transferrin, glucose)	Active	[47,48,57,64,69,82,108,109,110,111,112,113,114,115,116,117,118,119,120,121]
Vitamins (folate, riboflavin)	Active	[95,96,97,98,99,100]
Polymers (poly-L-lactide; hyaluronic acid, cholanic acid)	Passive	[56,60]
Polypeptide (Polylysine)	Passive	[49,58,63,64,122,123,124,125,126]
Heparin	Passive	[55,62]
D-α tocopheryl polyethylene glycol 1000 succinate (TPGS)	Passive	[80]
Quaternary ammonium cations	Passive	[87]

### 4.3. Key Factors for an Efficient Imaging of NPs in Cells

Identification of tissue alterations or lesion characterization as well as the diagnosis and treatment of disease through NP-based imaging techniques requires functionalization with contrast agents to be followed with imaging devices. Different molecules with optical properties such as fluorophore and bioluminescent dyes, isotopes or chemical elements with high molecular weight have been investigated as contrast agents. It is noteworthy that a considerable part of the reviewed studies suggested a combinatorial usage of contrast agents as an effective strategy for multimodal in vitro and/or in vivo tracking, as it could allow the limitations found with the use of a single-tracking approach to be overcome [130]. For this purpose, several protocols for the surface modification of inorganic NPs with specific elements have been developed. Because of this, the chemical element in the core of the NP represents one contrast moiety, and the molecule used for surface functionalization with an additional label. another. As an example, bi-modal tracking with MRI and PA has been performed by adopting NPs with two different metallic elements, such as gold and iron oxide [45,46]. A combination of fluorophores and metallic gold and iron oxide contrast agents was successfully used for optical and MRI tracking [47,48,49,50,87,88]. A review of the surface modification moieties for in vitro and/or in vivo tracking of the NPs identified by our bibliographic survey is included in Table 2.

### 4.4. Key Factors for Tracking Therapeutic NPs through Imaging

Advances in the field of NP-based cell tracking have led to extraordinary progress, with several strategies being deployed to also track therapy delivery through imaging. Several therapeutic NPs that are capable of both self-reporting disease and/or tissue damage and delivering therapy have been developed to date and play a central role for the implementation of currently available diagnostic and therapeutic protocols [24]. As an example, therapy followed by imaging might be useful to test reactions in order to treat and identify patients in which therapy has an effect with the goal of providing personalized therapy for individual patients. Functionalization allowing the use of these NPs as therapeutic agents, referred to as therapeutic moieties consist of molecules managing the proliferative and inflammation status of the target cell.

Therapeutic moieties identified by the present literature survey include miRNA [68,69], siRNA [78,187], genes [164] and drugs or compounds with key roles in the modulation of cell proliferation and differentiation [66,67,79,80,104,146,166,188] (Table 3).

Some examples of the adoption of these moieties are listed as follows. The miRNA AntimiR-138 was shown to significantly promote the expression of osteogenesis-related genes and its conjugation to the NPs used to label the MSCs was found to efficiently enhance the osteogenic differentiation of transplanted MSCs and direct cranial bone regeneration in preclinical mouse models [69]. Similarly, Hosseinpour and colleagues adopted NPs functionalized with the miRNA -26a-5p to label MSCs to be transplanted for tissue regeneration purposes [68].

siRNAs targeting genes whose expression is enhanced in tumors, such as marker B-cell lymphoma-2 (BCL2) and baculoviral IAP repeat-containing 5 (BIRC5) as well as genes with regulatory roles in sustaining cancer cell proliferation and migration, were used for NP functionalization and were found to selectively blockade the cancer cell proliferation of oral [115] and glioblastoma [78] cancer cell models.

NP functionalization with a model gene, consisting of a plasmid containing the green fluorescent protein, enabled gene transfer to the hMSCs showing the potential use of NPs for gene therapy delivery [121].

Chemotherapy drugs such as doxorubicin and methotrexate as well as molecules with the ability to modulate cell proliferation and differentiation events such as dexamethasone, betamethasone, caspase inhibitors, ceramide, oleanolic acid, emodin, heparin, aspirin, curcumin and sulforaphane were also adopted as therapeutic moieties for NP functionalization and found to be effective in (1) blocking cancer cell proliferation in lymph node [183] glioma [188], ovarian [146], hepatocellular [189], liver [79,80], cervical [95], pancreatic ductal [190] tumor models; (2) exerting anti-inflammatory actions [66]; (3) directing stem cell differentiation [62]; and (4) targeting apoptotic events of cerebral ischemia [166].

Importantly, in all the mentioned studies the functionalization of NPs with tracking moieties also working as contrast agents enabled a solid strategy for monitoring the effect of NP-cell based therapy to be set up.

**Table 3:** NP surface moieties with therapeutic effects.

Therapeutic Moieties	References
miRNA	[68,69]
siRNA	[78,187]
Drugs (doxorubicin, dexamethasone, betamethasone, methotrexate, caspase inhibitors, ceramide, oleanoic acid, emodin, heparin, aspirin, curcumin and sulforaphane)	[66,67,79,95,103,132,147,167,189]
Gene	[164]

**Figure 4:**
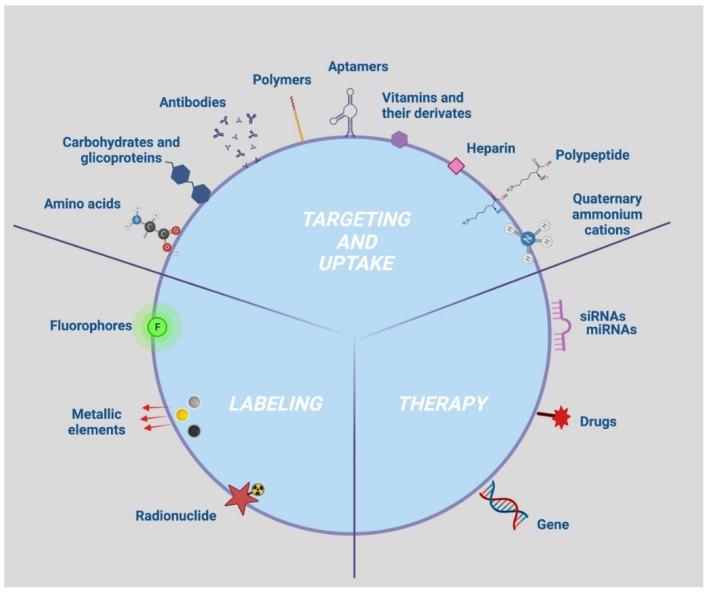
Multimodal NP surface modifications applied for enhancing targeting, uptake, tracking, and therapy. Identification of different elements useful to improve labeling, targeting, therapy and cellular uptake. Figure created with BioRender. Available online: https://biorender.com/ (accessed on 25 March 2022).

The surface modifications for NP delivery, diagnostic imaging and tracking of therapeutic effects are summarized in Figure 4.

## 5. Approaches and Devices for In Vitro, In Vivo and Ex Vivo Tracking of NP Delivery and for Assessing NP Stability

### 5.1. In Vitro and In Vivo Tracking of NP Delivery

Based on the present literature review, fluorophore labeling moieties have been found to be suitable for both in vitro and in vivo tracking. Conversely, labeling with radionuclides was only applied for in vivo tracking.

In detail, optical imaging instruments such as confocal scanning, fluorescence microscopy and flow cytometry have been adopted for in vitro fluorophore tracking whereas the fluorescence imaging devices such as the NIR fluorescence IVIS Spectrum system, fluorescence molecular tomography imaging and single- and two-photon fluorescence imaging have been applied for in vivo fluorescence tracking. Conversely, radionuclide-labeled NP monitoring has been achieved in vivo by means of nuclear molecular imaging tools such as single photon emission computed tomography (SPECT) and positron emission tomography (PET).

The multimodality of imaging has also been investigated by incorporating labeled NPs with cells bearing ectopic expression of the luciferase gene [43,115,170] and provided a non-invasive and more effective imaging technology to track transplanted cells [43,170] and stem cell homing [115].

The multilabeling system represents an advantageous approach as it provides different molecules which can act as contrast agents to be distinctively used for in vitro and in vivo NP delivery characterization. Importantly, multilabeling possibilities rely on the definition of the NP material and type. Indeed, differently from polymeric and lipid-based NPs, the tracking of inorganic NPs also takes advantage of the intrinsic attitude of the constituent core element to work as a contrast agent. In that case, both the metallic core and the surface labeling moiety enable in vitro and in vivo tracking of NP delivery.

**Figure 5:**
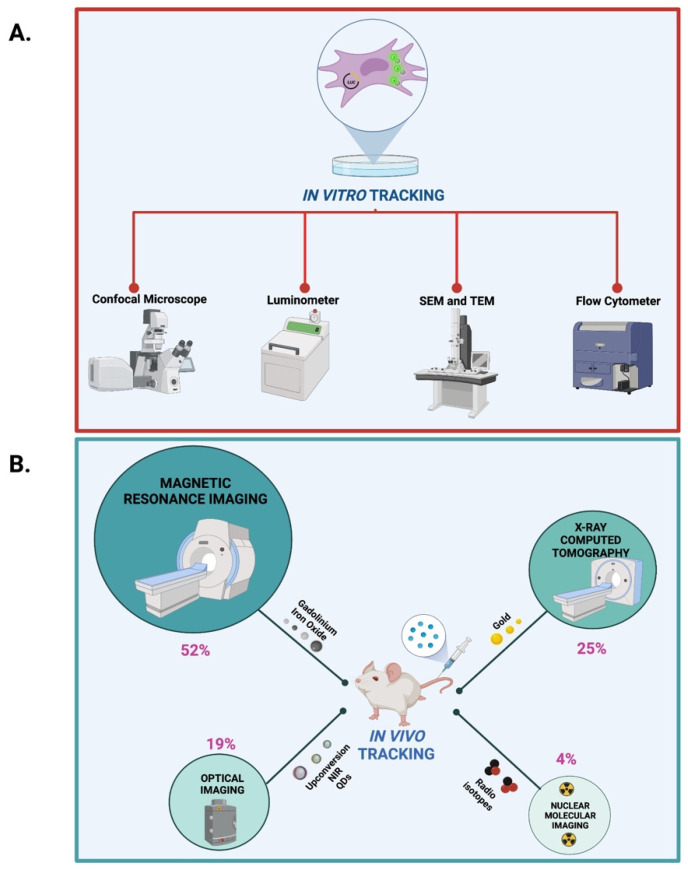
Methods and devices for in vivo and in vitro imaging of NPs. (**A**) In vitro tracking of NPs. (**B**) In vivo tracking of NPs. Percentage (%) refers to different types of imaging tools for NP tracking based on our literature survey. Figure created with BioRender. Available online: https://biorender.com/ (accessed on 25 March 2022).

In vitro tracking of the core element can be achieved by means of transmission electron microscopy (TEM) and scanning electron microscopy (SEM), whereas NP surface-labeling moieties such as fluorophores and cell-labeling moieties such as luciferin can be followed in vitro by using confocal laser microscope and luminometer, respectively (Figure 5A and Supplementary Materials Table S3). On the other hand, in vivo tracking of the core element can be accomplished with MRI and tomography imaging such as computed tomography (CT), microcomputed tomography (µCT), photoacoustic tomography (PA) and X-ray fluorescence tomography (XFT) imaging.

It is noteworthy that, according to NP classification, our literature survey identified the MRI (52%), followed by CT (25%), optical (19%) and radionuclide (4%) imaging as the most used in vivo imaging devices (Figure 5B and Supplementary Materials Table S4).

Two different aspects bind the choice of the imaging technique: (I) the tissue to be targeted and (II) the sensitivity of the technique.

MRI represents the imaging method with the best soft tissue contrast, providing multiplanar capability without the use of ionizing radiation. It has emerged as the preferred modality for evaluating soft tissue masses and vascular structures, and it has been applied for checking soft tumors and inflammatory foci [191]. Iron oxide and gadolinium have been described as the most used contrast agents for MRI. While an MRI takes excellent pictures of soft tissue and blood vessels, a CT scan shows hard tissues much better, so it is often used to image bone structures [192]. Gold elements represent the elective contrast agent of CT. Many studies have shown that due to their strong X-ray absorption coefficient, good colloidal stability and biocompatibility, sustained contrast, shape and size controllability, surface modifiability, and few negative impacts on the marked cells, gold nanomaterials are ideal CT nano tracers for tracking [44]. As examples, with the use of this strategy, it was possible to demonstrate non-invasive cell tracking towards tumor sites [118], understand the fate of transplanted mesenchymal stem cells in certain diseases such as pulmonary fibrosis [43], and allow non-invasive and long-term CT monitoring of mesenchymal stem cells in tissue repair [123].

In contrast to MRI and CT, the two most used and clinically approved techniques for in vivo tracking, optical imaging is well suited for non-clinical use. However, it retains the potential for clinical application as it could exploit an enormous range of contrast agents such as molecules with fluorescent, near infrared or bioluminescent properties, that provide information about the structure and function of tissues ranging from single cells to entire organisms. An additional benefit of optical imaging that is often underexploited is its ability to acquire data at high speeds; a feature that enables it to not only observe static distributions of contrast, but to probe and characterize dynamic events related to physiology, disease progression and acute interventions in real time [193].

A few studies applied nuclear molecular imaging for NP tracking in vivo. Although this technique provides highly sensitive and quantitative imaging and shows good tissue penetration depth, it is limited by its low spatial resolution [194]. This was reflected in the small number of research articles collected by our bibliographic approach.

Apart from these limitations, nuclear molecular imaging holds great potential for preclinical research and clinical applications as it might be a valuable tool granting long-term monitoring of cells, as well as the observation of cell clearance from various organs and homing at the tumor site.

NPs can be labeled with several radionuclides and have been proposed as innovative tools for imaging with single-photon emission computerized tomography (SPECT) and positron emission tomography (PET) and enhanced radioisotope therapy for several cancer types with limited toxicity [195].

Radionuclide-labeled NPs mostly characterized for diagnostic imaging and/or therapy purposes include technetium-99m (99mTc) [196], fluorine-18 (18F) [197], gallium-68 (68Ga) [198], indium-111 (111In) [199], and lutetium-177 (177Lu) [200].

The imaging and therapeutic applications of an isotope depend on its particle emission. Gamma (γ)-ray emitters such as 99mTC, 111In and positron emitters such as 18F, 68Ga are commonly used for diagnostic purposes using SPECT and PET imaging, respectively, and β- emitters such as 177Lu are mostly, but not exclusively, used for radionuclide therapy.

An appealing strategy that has proved beneficial for cell imaging with radionucleotides was its adoption in combination with other imaging tools such as those with magnetic and/or X-ray properties to combine the advantages of different imaging modalities and minimize the limitations [201]. Of note, multimodality imaging systems such as SPECT/MRI, PET/MRI, SPECT/CT, and PET/CT are currently under investigation. As some examples, the conjugation of 99mTc with silica [202], iron oxide [203] or gold [204] NPs was exploited for SPECT/MRI or SPECT/CT imaging of tumors in preclinical mouse models. Likewise, the conjugation of 18F with iron oxide NP was found effective for dual imaging with PET/MRI of stem cell biodistribution [205] as well as for monitoring anti-angiogenic therapeutic effects in breast cancer xenograft mouse models [206] and diagnosis of rectal cancer in clinics [207]. Importantly, since PET/MRI systems have become operational worldwide, several imaging protocols involving the use of 18F for whole body scanning in oncology are currently under standardization for patient management in clinics [208].

Radionuclide imaging for tumor diagnosis was also performed by adopting NPs conjugated with radiopharmaceutical formulations of the (γ)-ray emitter 111In (such as indium-111-oxine) allowing the monitoring of migration and homing of intravenously given stem cell-bearing NPs to the tumor location [55,88,180].

Advantageous strategies for simultaneous diagnostics and therapy are also being characterized. In this context, a promising strategy is the pairing of radionuclides of different elements [195]. As an example, 68Ga for PET imaging and 177Lu as β-emitter for a therapy couple have been investigated as a radiopharmaceutical tool to face prostate [181] colon [182], lymph node [183,184,185] and glioblastoma [186] cancers.

Overall, based on these premises, it would be helpful to find a compromise between invasiveness and diagnostic resolution to improve actual NP tracking methods.

### 5.2. Ex Vivo NP Tracking of NP Delivery

Specific ex vivo protocols have been developed to verify and quantify NP uptake on tissue and cell specimens prior to being applied for in vivo protocols. Several histochemical dyes can be adopted for that purpose (Table 4). Inorganic NP uptake has been reported to be obtained by using Prussian blue or alizarin red dyes as their ability to stain ferric ions and minerals, respectively. Conversely, Oil Red O dye, with its affinity to lipids, was successfully applied for staining lipidic NPs.

Moreover, ex vivo checking, and quantification of cell uptake was performed by coating NPs with PI or Nile red dye. As example, NP coating with PI was successfully adopted to detect cells incorporating gold NPs through flow cytometry [118], whereas Nile red labeling of hybrid iron oxide/polymeric NPs was used to check in vitro uptake into macrophages [89].

### 5.3. NP Stability Assessment

Another key element influencing the performance of any NP-based cell tracking strategy is the stability of the NPs. Any NP formulation to be applied for tracking purposes needs to preserve a particular nanostructure property including aggregation, composition, shape, size, and surface chemistry.

Different environmental stresses such as extended storage, pH and mineral composition, thermal processing, freeze–thaw cycling, dehydration, mechanical stress and light exposure have been described as responsible for influencing NP stability [29].

The ability to measure the stability of the NP formulation prior to utilization for tracking purposes thus represents a key step to be pursued. Methods that provide quantitative metrics for measuring and modeling nanoparticle stability in terms of core composition, shape, size, and surface chemistry are distinguished by their physical- and chemical- [226,227] based approaches and are summarized in Table 5.

## 6. NP Uptake Strategies and Mechanisms

The effects of the physicochemical properties of NPs, such as size, shape, charge and surface chemistry, are crucial for determining the cellular uptake mechanism and the NP function exerted upon the cells [228]. Importantly, knowledge of the underlying mechanisms involved in cellular NP incorporation is found to be relevant for the fate of the NPs and their cytotoxicity [90], as well as for safe and efficient therapeutic applications [229]. Our bibliographic survey identified the endocytosis mechanism as the most representative for the internalization of NPs (97%). Endocytosis can be accomplished through two main mechanisms: phagocytosis and pinocytosis. Phagocytosis is the preferred uptake for larger nanoparticles (≥100 nm), while pinocytosis dominates the uptake of smaller nanoparticles (≤100 nm) [230,231]. Of note, pinocytosis can be subdivided into clathrin-mediated and caveolae-dependent endocytosis, to date, considered as some of the most important mechanisms for NP uptake [229,230]. However, since the variation in endocytic mechanisms depends on cell and NP types, it is still challenging to generalize the current findings [229].

Another key aspect to be considered when testing cell uptake efficiency is the NP incubation time, which is influenced by the type and density of the cultured cells [232] and the shape, size and concentration of the NPs [233,234]. Our literature survey identified 24 h as the most validated incubation time for different types of cell target. However, it has been also proved that the addition of some compounds to NPs might shortening the time required for cell uptake: consistently, Mishra and colleagues showed that the use of protamine sulfate for inorganic iron oxide NP coating reduced the incubation time to 6 h in MSCs [59].

Cell density also influences NP uptake; the most suitable cell density reported in the reviewed studies refers to 10^4^ cells/cm^2^ with high variability defined by the type of cells adopted for the uptake assay. However, several uptake protocols are currently under investigation to increase the quantity of the recovered cells with the aim of having a bulk cell preparation to be used for in vivo NP delivery and tracking. Even in this case, tracking sensitivity reflects the resolution of the imaging technique applied; thus, it was not possible to identify a reference interval for cell number to be injected in in vivo mouse models. Generally, based on the reviewed studies, in vitro protocols pointed to the recovery of at least totally 10^6^ cells. Likewise, NP concentration parameters were highly fluctuating and were strictly correlated to the NP architecture and size.

Alternative strategies identified with the presented literature review include the adoption of electroporation and transfection accounting, respectively, for about 2% and 1% of the total citations. Electroporation was reported to be useful in assisting the passage of NPs larger than 100 nm through the cell membrane [44] or to enhance the quantity of incorporated NPs [235].

**Figure 6:**
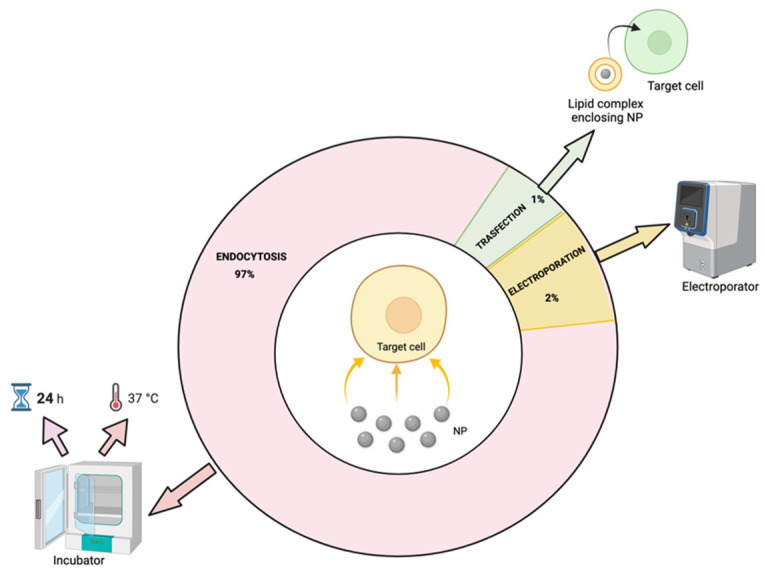
NP uptake mechanisms and strategies. Figure created with BioRender. Available online: https://biorender.com/ (accessed on 25 March 2022).

Furthermore, the adoption of transfectant agents such as commercially available lipofectamine formulations has been proved to increase uptake efficiency. Accordingly, Wan et al. designed a new approach involving the surface modification of gold NPs with layers of silica and Transfectin 3000 (TS) to reduce cytotoxicity and improve the incorporation in bone marrow-derived stem cells (BMSCs) [236]. Uptake strategies identified in the literature survey are summarized in Figure 6.

## 7. Cell Types Suitable for Safe NP Delivery

As previously mentioned, improved NP delivery can be achieved by using cells as the vehicle for NPs. Based on our literature survey, different types of cells have been investigated in the last decade as potential carriers for an efficient NP release.

**Figure 7:**
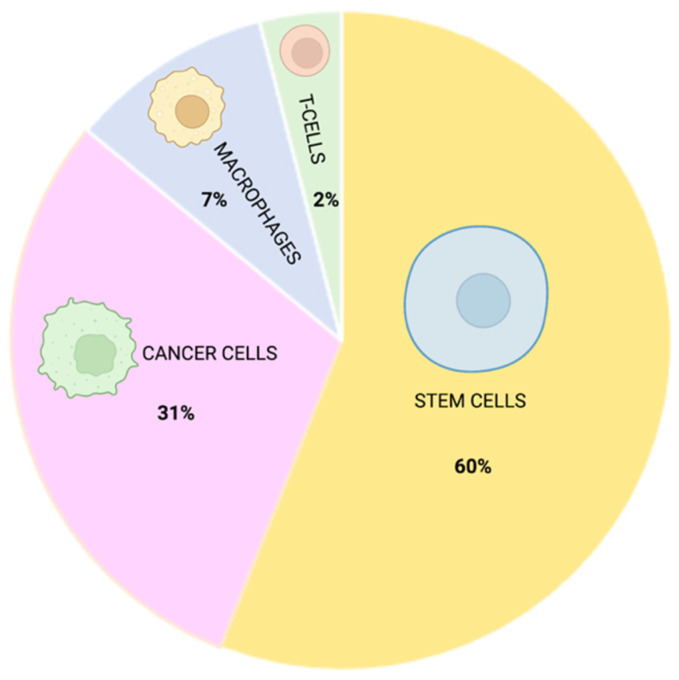
Cell sources adopted for efficient and safe NP delivery. Cell type subcategories and their relative percentage of utilization identified by the literature survey. Figure created with BioRender. Available online: https://biorender.com/ (accessed on 25 March 2022).

Stem cells, with their intrinsic ability to home in on cancer or injury sites, represent the most suitable cell type source allowing NP delivery, with a 60% of the reviewed studies investigating their potential for biomedical application for cancer and regenerative medicine purposes. Conversely, a smaller percentage of studies (31%), focusing on the development of clinical protocols for precision cancer medicine, adopted cancer cells as the target of NP delivery. Only a few original research articles, pointing to the elucidation of inflammatory homing mechanisms, used other cell sources such as macrophages (7%) or T cells (2%) (Figure 7).

### 7.1. Stem Cells

Stem cell homing mechanisms are currently being investigated to provide further insights for their potential use as vehicles for contrast agents to be detected with currently available imaging techniques to improve the diagnosis and therapy of many diseases [237].

Different subtypes of stem cells were identified by reviewing our bibliographic dataset. In detail, mesenchymal stem cells (MSCs) accounted for 80% of the total citations using stem cells, 6% involved the use of adipose tissue-derived stem cells (ADSCs), and 14% instead involved the use of other different subtypes such as blastema cells [160], neuronal stem cells (NSC) [54], bone marrow dendritic cells (BMDCs) [55,219] and mouse embryonic stem cells (mESCs) [170].

Furthermore, stem cells derived from different species have been identified. Of note, many studies involved MSCs of human origin (84%), followed by those derived from rat (14%) and monkey (2%) species.

### 7.2. Cancer Cells

The most used cancer cell model identified with the present literature review is the HeLa cell line, with 44% of the total citations exploiting cancer cells for NP delivery purposes. HeLa was the first human cell line established in culture and has since become the most widely used human cell line in cancer research.

The choice of the cancer cell model relies on the type of cancer to be targeted. Thirteen percent of the studies focused their attention on the investigation of nanotechnology supporting hepatocellular carcinoma resolution by means of hepatocarcinoma-derived cell lines such as hepatoma cell line Hepa1–6 [91], HepG2 cells and HCa-F [80,100,136] and hepatocarcinoma cell line SMMC-7721 [111].

Cancer cell lines derived from glioma or glioblastoma, such as human GBM cell lines U-87 and U251 [78,180], U118 glioma [238] and F98 rat glioma [239] cells were represented by 13% of the total citations describing the application of cancer cell lines.

Similarly, 10% of studies aimed to identify therapeutic approaches against breast cancer with the MDA-MB-231 human breast cancer cell line [179,215,221] and breast cancer 4T1 cell line [162].

Ten percent of studies have investigated efficient protocols to cure lung cancer by means of lung cancer cell models such as A529, A549, NCI H441 and A2780 [134,141,152,169].

To a lesser extent, studies have been carried out with cell lines derived from pancreatic cancer such as Panc-1 and MIA Paca-2 cells [190] and human pancreatic cancer cell line BxPc-3 [94] and also derived from gastric cancer such as human gastric cancer cells (MGC80-3) [132,138], both of these cell types account for 5% of the total citations concerning the use of cancer cell line models for NP delivery.

### 7.3. Macrophages

Macrophage-bearing NPs have been adopted mostly for tracking. Macrophages were used to evaluate uptake of nanoparticles using imaging techniques [147,240] to investigate the metabolic conversion and elimination of nanoparticles [218] and since these macrophages are able to migrate to sites of inflammation [112], they have been labeled with NPs and their distribution assessed by clinically relevant imaging techniques [89,109,116,222]. Although little is known about NP delivery efficiency of macrophages, this cell source might represent a promising tool for inflammation-targeting purposes in the fields of both regenerative medicine and cancer diseases [241].

### 7.4. T Cells

T cells have been shown to kill malignant cells in vitro and in vivo, therefore, several studies have labeled these cells with gold NPs or SPIONs to follow their fate on CT or MRI. For example, T cells were transduced to express a melanoma-specific T cell, and then computed tomography analysis was performed, once labeled with gold NPs. CT efficiently detected the accumulation of these transduced and labeled T cells at the tumor site, leading to tumor regression [118]. Another study preferred to label T cells with ultrasmall SPIOs to follow their fate through MRI [242]. Overall, although T cells encapsulating NPs have been poorly characterized to date, their use in nanomedicine deserves attention for improving the efficacy of T-cell based immunotherapy [172].

## 8. Conclusions and Outlook for Effective NP-Based Cell Tracking

Issues determining the effectiveness of NP-assisted cell tracking such as NP design, material properties and functionalization with specific targeting, labeling (tracking) and/or uptake-enhancing moieties have been identified and almost exhaustively characterized during the last decade. Nevertheless, an in-depth analysis of the features relating to NPs and the interaction dynamics with the target body area, which in turn is influenced by the cell to be targeted, the NP loading time and concentration, still needs to be achieved.

The nexus of NPs with the biology is currently an area of intense study in the field of NP-based cell tracking as the success of NP incorporation into the cell and consequently the success of direct cell labeling in terms of non-invasive long-term targeted monitoring through imaging depends on the type of biological response triggered by NP uptake.

The investigation of mechanisms determining the efficacy of the active incorporation of NPs by the cell holds a prominent role in NP-assisted cell tracking as it keeps the potential clinical traceability for a targeted, sensitive, and long monitoring of cells with the current clinically approved imaging devices improving the performance of the currently used iodinated, barium or gadolinium-based contrast agents [14].

Much effort has been made to better characterize the biological cues of NP–cell dialogue with the aim of improving uptake and retention mechanisms to be exploited for direct cell tracking; despite its simple and straightforward nature, this approach has several drawbacks which deserve further investigation to improve their effective clinical use.

Specifically, the biggest limitation of any direct labeling method is the failure in the assessment of cell viability and division damping retention and the right delivery of NPs. Firstly, if the cells die, the NPs may be retained at the target site, being taken up by macrophages consequently leading to erroneous detection of cells. The NPs may also dissociate from the cells through efflux and exocytosis, leading to false quantification and monitoring of free labels instead of the cells of interest. Secondly, cell division dilutes the number of NPs, affecting the sensitivity of the detection of daughter cells and limiting the long-term tracking of labeled cells [243]. Overall, these aspects can be managed by deepening the characterization of cell-fate mechanisms and how they are modulated by NP incorporation.

**Figure 8:**
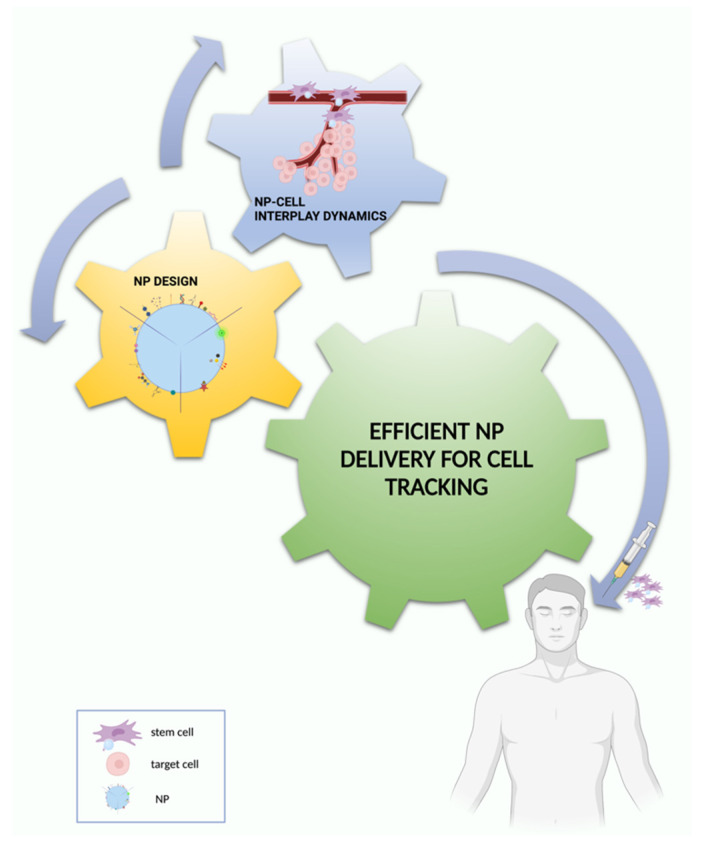
Essential ingredients for effective NP–cell tracking. NP design, cellular models and interaction mechanisms between NPs and cargo stem cells and/or target cells are key elements influencing NP-based cell tracking and delivery performances. Figure created with BioRender. Available online: https://biorender.com/ (accessed on 25 March 2022).

Considering these premises, the definition of the impact of NP physicochemical properties on cell internalization mechanisms, together with the characterization of biological cell response might pave the way for effective NP-delivery solutions for cell tracking, finally providing future perspectives for a bench to bedside translation (Figure 8).

## Figures and Tables

**Table 2:** Moieties for labeling. NPs can be functionalized with labeling moieties allowing both in vitro and in vivo NP tracking delivery.

Moieties for Labeling	In Vitro	In Vivo
Fluorophores (fluorescein, rhodamine, squaraine, acridine, DY674, cianine, AxHD dye, SR-FLIVO dye, IR780, selenium, NIR dyes, becon probe, RuBpy, IRGD, I-BODIPY, Fluo4, PKH26)	[43,52,53,54,66,74,75,83,95,98,105,107,115,120,123,131,132,133,134,135,136,137,138,139,140,141,142,143,144,145,146,147,148,149,150,151,152,153,154,155,156,157,158,159,160,161,162,163,164,165,166,167,168,169,170,171,172,173,174,175,176]	[43,52,54,61,66,83,95,96,105,115,123,157,158,160,161,162,163,164,165,166,167,168,169,170,171,172,173,174,175,176,177,178,179]
Radionuclides (Indium-111; Gallium-68; Lutetium-177)	*-*	[55,88,180,181,182,183,184,185,186]

**Table 4:** Dyes used for ex vivo quantification of NP uptake. Dyes can react with elements characterizing the NPs or can be applied for NP coating.

Dye	Type	References
Histochemical staining	Prussian blue	[48,49,51,57,58,59,64,65,88,109,114,116,117,124,127,138,154,155,160,177,187,189,209,210,211,212,213,214,215,216,217,218,219,220,221,222,223,224]
	Oil Red O	[58,225]
	Alizarin red	[225]
Dyes for NP coating	Propidium iodide (PI)	[118]
	Nile red	[89]

**Table 5:** Key parameters defining NP stability and strategies to determine stability preservation.

NP Stability	Definition	Approaches Used for Characterization of NP Stability	
		Physical	Chemical
Aggregation	Preservation of NPs upon collisions	Dynamic light scattering	Single particle inductively coupled plasma mass spectrometry UV–visible spectroscopy
Core Composition	Unchanged chemistry of the core during the use	X-ray diffraction	Single particle inductively coupled plasma mass spectrometry UV–visible spectroscopy Surface-enhanced Raman scattering X-ray photoelectron spectroscopy Energy dispersive X-ray
Shape	Preservation of NP architecture during the use	Transmission electron microscopy Scanning electron microscopy X-ray diffraction Atomic force microscopy	Single particle inductively coupled plasma-mass spectrometry UV–visible spectroscopy
Size	Preservation of NP dimension during use or storage	Dynamic light scattering Scanning electron microscopy Transmission electron microscopy Small-angle X-ray scattering Atomic force microscopy	Single particle inductively coupled plasma-mass spectrometry UV–visible spectroscopy
Surface chemistry	Preservation of the native surface functionality	Low energy ion scattering X-ray photoelectron spectroscopy	Single particle inductively coupled plasma-mass spectrometry UV–visible spectroscopy Surface-enhanced Raman scattering X-ray photoelectron spectroscopy Energy dispersive X-ray

## Data Availability

Not applicable.

## Supplementary Materials

The following supporting information can be downloaded at: https://www.mdpi.com/article/10.3390/nano12091414/s1, Table S1: Clinical trial studies investigating the use of magnetic NPs for cell tracking; Table S2: Database of citations exploring direct cell tracking approaches through imaging; Table S3: General operational principles of the technologies used for in vitro cell tracking; Table S4: General operational principles of the technologies used for in vivo cell tracking. References [244,245,246,247] are cited in the supplementary materials.

## References

[1] Zimmer C., Zhang B., Dufour A., Thebaud A., Berlemont S., Meas-Yedid V., Marin J.-C.O. (2006). On the digital trail of mobile cells. IEEE Signal Process. Mag..

[2] Meijering E., Smal I., Danuser G. (2006). Tracking in molecular bioimaging. IEEE Signal Process. Mag..

[3] Meijering E., Dzyubachyk O., Smal I., van Cappellen W.A. (2009). Tracking in cell and developmental biology. Semin. Cell Dev. Biol..

[4] Dorn J.F., Danuser G., Yang G. (2008). Computational processing and analysis of dynamic fluorescence image data. Methods Cell Biol..

[5] Jaqaman K., Danuser G. (2009). Computational image analysis of cellular dynamics: A case study based on particle tracking. Cold Spring Harb. Protoc..

[6] Rohr K., Godinez W.J., Harder N., Wörz S., Mattes J., Tvaruskó W., Eils R. (2010). Tracking and quantitative analysis of dynamic movements of cells and particles. Cold Spring Harb. Protoc..

[7] Ray S.S., Bandyopadhyay J. (2021). Nanotechnology-enabled biomedical engineering: Current trends, future scopes, and perspectives. Nanotechnol. Rev..

[8] McNamara K., Tofail S.A.M. (2017). Nanoparticles in biomedical applications. Adv. Phys. X.

[9] Bhirde A., Xie J., Swierczewska M., Chen X. (2011). Nanoparticles for cell labeling. Nanoscale.

[10] Arranz A., Ripoll J. (2015). Advances in optical imaging for pharmacological studies. Front. Pharmacol..

[11] Ni J.-S., Li Y., Yue W., Liu B., Li K. (2020). Nanoparticle-based Cell Trackers for Biomedical Applications. Theranostics.

[12] Van Rijt S., Habibovic P. (2017). Enhancing regenerative approaches with nanoparticles. J. R. Soc. Interface.

[13] Zhang Y., Li M., Gao X., Chen Y., Liu T. (2019). Nanotechnology in cancer diagnosis: Progress, challenges and opportunities. J. Hematol. Oncol..

[14] Bernsen M.R., Guenoun J., van Tiel S.T., Krestin G.P. (2015). Nanoparticles and clinically applicable cell tracking. Br. J. Radiol..

[15] Zhang M., Cheng S., Jin Y., Zhang N., Wang Y. (2021). Membrane engineering of cell membrane biomimetic nanoparticles for nanoscale therapeutics. Clin. Transl. Med..

[16] Thomas S.C., Kim J.-W., Pauletti G.M., Hassett D.J., Kotagiri N. (2022). Exosomes: Biological Pharmaceutical Nanovectors for Theranostics. Front. Bioeng. Biotechnol..

[17] Accomasso L., Gallina C., Turinetto V., Giachino C. (2016). Stem Cell Tracking with Nanoparticles for Regenerative Medicine Purposes: An Overview. Stem Cells Int..

[18] Huang X., Zhang F., Wang H., Niu G., Choi K.Y., Swierczewska M., Zhang G., Gao H., Wang Z., Zhu L. (2013). Mesenchymal stem cell-based cell engineering with multifunctional mesoporous silica nanoparticles for tumor delivery. Biomaterials.

[19] Perrin J., Capitao M., Mougin-Degraef M., Guérard F., Faivre-Chauvet A., Rbah-Vidal L., Gaschet J., Guilloux Y., Kraeber-Bodéré F., Chérel M. (2020). Cell Tracking in Cancer Immunotherapy. Front. Med..

[20] Mu Q., Wang H., Zhang M. (2017). Nanoparticles for imaging and treatment of metastatic breast cancer. Expert Opin. Drug Deliv..

[21] Ruiz-Garcia H., Alvarado-Estrada K., Krishnan S., Quinones-Hinojosa A., Trifiletti D.M. (2020). Nanoparticles for Stem Cell Therapy Bioengineering in Glioma. Front. Bioeng. Biotechnol..

[22] Tiet P., Berlin J.M. (2017). Exploiting homing abilities of cell carriers: Targeted delivery of nanoparticles for cancer therapy. Biochem. Pharmacol..

[23] Mitchell M.J., Billingsley M.M., Haley R.M., Wechsler M.E., Peppas N.A., Langer R. (2021). Engineering precision nanoparticles for drug delivery. Nat. Rev. Drug Discov..

[24] Zavaleta C., Ho D., Chung E.J. (2018). Theranostic Nanoparticles for Tracking and Monitoring Disease State. SLAS Technol..

[25] Mason E.E., Mattingly E., Herb K., Śliwiak M., Franconi S., Cooley C.Z., Slanetz P.J., Wald L.L. (2021). Concept for using magnetic particle imaging for intraoperative margin analysis in breast-conserving surgery. Sci. Rep..

[26] Wei Q., Arami H., Santos H.A., Zhang H., Li Y., He J., Zhong D., Ling D., Zhou M. (2021). Intraoperative Assessment and Photothermal Ablation of the Tumor Margins Using Gold Nanoparticles. Adv. Sci..

[27] Onishi T., Mihara K., Matsuda S., Sakamoto S., Kuwahata A., Sekino M., Kusakabe M., Handa H., Kitagawa Y. (2022). Application of Magnetic Nanoparticles for Rapid Detection and In Situ Diagnosis in Clinical Oncology. Cancers.

[28] Najahi-Missaoui W., Arnold R.D., Cummings B.S. (2020). Safe Nanoparticles: Are We There Yet?. Int. J. Mol. Sci..

[29] Phan H.T., Haes A.J. (2019). What Does Nanoparticle Stability Mean?. J. Phys. Chem. C Nanomater. Interfaces.

[30] Núñez C., Estévez S.V., Del Pilar Chantada M. (2018). Inorganic nanoparticles in diagnosis and treatment of breast cancer. J. Biol. Inorg. Chem. JBIC Publ. Soc. Biol. Inorg. Chem..

[31] Soenen S.J., Parak W.J., Rejman J., Manshian B. (2015). (Intra)cellular stability of inorganic nanoparticles: Effects on cytotoxicity, particle functionality, and biomedical applications. Chem. Rev..

[32] Jiao M., Zhang P., Meng J., Li Y., Liu C., Luo X., Gao M. (2018). Recent advancements in biocompatible inorganic nanoparticles towards biomedical applications. Biomater. Sci..

[33] Zielińska A., Carreiró F., Oliveira A.M., Neves A., Pires B., Venkatesh D.N., Durazzo A., Lucarini M., Eder P., Silva A.M. (2020). Polymeric Nanoparticles: Production, Characterization, Toxicology and Ecotoxicology. Molecules.

[34] Ma D. (2019). Hybrid Nanoparticles. Noble Metal-Metal Oxide Hybrid Nanoparticles.

[35] García-Pinel B., Porras-Alcalá C., Ortega-Rodríguez A., Sarabia F., Prados J., Melguizo C., López-Romero J.M. (2019). Lipid-Based Nanoparticles: Application and Recent Advances in Cancer Treatment. Nanomaterials.

[36] Singh P., Bodycomb J., Travers B., Tatarkiewicz K., Travers S., Matyas G.R., Beck Z. (2019). Particle size analyses of polydisperse liposome formulations with a novel multispectral advanced nanoparticle tracking technology. Int. J. Pharm..

[37] Vitorino C., Carvalho F.A., Almeida A.J., Sousa J.J., Pais A.A.C.C. (2011). The size of solid lipid nanoparticles: An interpretation from experimental design. Colloids Surf. B Biointerfaces.

[38] Khosa A., Reddi S., Saha R.N. (2018). Nanostructured lipid carriers for site-specific drug delivery. Biomed. Pharmacother. Biomed. Pharmacother..

[39] Fan Y., Marioli M., Zhang K. (2021). Analytical characterization of liposomes and other lipid nanoparticles for drug delivery. J. Pharm. Biomed. Anal..

[40] Mehnert W., Mäder K. (2012). Solid lipid nanoparticles. Adv. Drug Deliv. Rev..

[41] Bulte J.W.M., Daldrup-Link H.E. (2018). Clinical Tracking of Cell Transfer and Cell Transplantation: Trials and Tribulations. Radiology.

[42] Sun Y., Lu Y., Yin L., Liu Z. (2020). The Roles of Nanoparticles in Stem Cell-Based Therapy for Cardiovascular Disease. Front. Bioeng. Biotechnol..

[43] Bao H., Xia Y., Yu C., Ning X., Liu X., Fu H., Chen Z., Huang J., Zhang Z. (2019). CT/Bioluminescence Dual-Modal Imaging Tracking of Mesenchymal Stem Cells in Pulmonary Fibrosis. Small.

[44] Huang J., Huang J.H., Bao H., Ning X., Yu C., Chen Z., Chao J., Zhang Z. (2020). CT/MR Dual-Modality Imaging Tracking of Mesenchymal Stem Cells Labeled with a Au/GdNC@SiO_2_ Nanotracer in Pulmonary Fibrosis. ACS Appl. Bio Mater..

[45] Zhang H., Wang Z.-J., Wang L.-J., Li T.-T., He S., Li L.-P., Li X.-Y., Liu S.-J., Li J.-D., Li S.-J. (2019). A dual-mode nanoparticle based on natural biomaterials for photoacoustic and magnetic resonance imaging of bone mesenchymal stem cells in vivo. RSC Adv..

[46] Chen P.-J., Kang Y.-D., Lin C.-H., Chen S.-Y., Hsieh C.-H., Chen Y.-Y., Chiang C.-W., Lee W., Hsu C.-Y., Liao L.-D. (2015). Multitheragnostic Multi-GNRs Crystal-Seeded Magnetic Nanoseaurchin for Enhanced In Vivo Mesenchymal-Stem-Cell Homing, Multimodal Imaging, and Stroke Therapy. Adv. Mater..

[47] Vaags A.K., Gartley C.J., Halling K.B., Dobson H., Zheng Y., Foltz W.D., Dick A.J., Kruth S.A., Hough M.R. (2011). Migration of cells from the yolk sac to hematopoietic tissues after in utero transplantation of early and mid gestation canine fetuses. Transplantation.

[48] Geburek F., Mundle K., Conrad S., Hellige M., Walliser U., van Schie H.T.M., van Weeren R., Skutella T., Stadler P.M. (2016). Tracking of autologous adipose tissue-derived mesenchymal stromal cells with in vivo magnetic resonance imaging and histology after intralesional treatment of artificial equine tendon lesions—A pilot study. Stem Cell Res. Ther..

[49] Qin J.-B., Li K.-A., Li X.-X., Xie Q.-S., Lin J.-Y., Ye K.-C., Jiang M.-E., Zhang G.-X., Lu X.-W. (2012). Long-term MRI tracking of dual-labeled adipose-derived stem cells homing into mouse carotid artery injury. Int. J. Nanomed..

[50] Meng Y., Shi C., Hu B., Gong J., Zhong X., Lin X., Zhang X., Liu J., Liu C., Xu H. (2017). External magnetic field promotes homing of magnetized stem cells following subcutaneous injection. BMC Cell Biol..

[51] Wu C., Li J., Pang P., Liu J., Zhu K., Li D., Cheng D., Chen J., Shuai X., Shan H. (2014). Polymeric vector-mediated gene transfection of MSCs for dual bioluminescent and MRI tracking in vivo. Biomaterials.

[52] Moonshi S.S., Zhang C., Peng H., Puttick S., Rose S., Fisk N.M., Bhakoo K., Stringer B.W., Qiao G.G., Gurr P.A. (2018). A unique 19F MRI agent for the tracking of non phagocytic cells in vivo. Nanoscale.

[53] Zhang Q., Nie J., Xu H., Qiu Y., Li X., Gu W., Tang G., Luo J. (2017). Fluorescent microspheres for one-photon and two-photon imaging of mesenchymal stem cells. J. Mater. Chem. B.

[54] Li D., Yan X., Hu Y., Liu Y., Guo R., Liao M., Shao B., Tang Q., Guo X., Chai R. (2019). Two-Photon Image Tracking of Neural Stem Cells via Iridium Complexes Encapsulated in Polymeric Nanospheres. ACS Biomater. Sci. Eng..

[55] Sehl O.C., Gevaert J.J., Melo K.P., Knier N.N., Foster P.J. (2020). A Perspective on Cell Tracking with Magnetic Particle Imaging. Tomography.

[56] Vernikouskaya I., Fekete N., Bannwarth M., Erle A., Rojewski M., Landfester K., Schmidtke-Schrezenmeier G., Schrezenmeier H., Rasche V. (2014). Iron-loaded PLLA nanoparticles as highly efficient intracellular markers for visualization of mesenchymal stromal cells by MRI. Contrast Media Mol. Imaging.

[57] Shahror R.A., Ali A.A.A., Wu C.-C., Chiang Y.-H., Chen K.-Y. (2019). Enhanced Homing of Mesenchymal Stem Cells Overexpressing Fibroblast Growth Factor 21 to Injury Site in a Mouse Model of Traumatic Brain Injury. Int. J. Mol. Sci..

[58] Mishra S.K., Khushu S., Singh A.K., Gangenahalli G. (2018). Homing and Tracking of Iron Oxide Labelled Mesenchymal Stem Cells After Infusion in Traumatic Brain Injury Mice: A Longitudinal In Vivo MRI Study. Stem Cell Rev. Rep..

[59] Mishra S.K., Khushu S., Gangenahalli G. (2017). Biological effects of iron oxide-protamine sulfate complex on mesenchymal stem cells and its relaxometry based labeling optimization for cellular MRI. Exp. Cell Res..

[60] Huang X., Zhang F., Wang Y., Sun X., Choi K.Y., Liu D., Choi J., Shin T.-H., Cheon J., Niu G. (2014). Design considerations of iron-based nanoclusters for noninvasive tracking of mesenchymal stem cell homing. ACS Nano.

[61] Shen W.-B., Plachez C., Tsymbalyuk O., Tsymbalyuk N., Xu S., Smith A.M., Michel S.L.J., Yarnell D., Mullins R., Gullapalli R.P. (2016). Cell-Based Therapy in TBI: Magnetic Retention of Neural Stem Cells In Vivo. Cell Transplant..

[62] Lee J., Jung M.J., Hwang Y.H., Lee Y.J., Lee S., Lee D.Y., Shin H. (2012). Heparin-coated superparamagnetic iron oxide for in vivo MR imaging of human MSCs. Biomaterials.

[63] Noorwali A., Faidah M., Ahmed N., Bima A. (2019). Tracking iron oxide labelled mesenchymal stem cells(MSCs) using magnetic resonance imaging (MRI) in a rat model of hepatic cirrhosis. Bioinformation.

[64] Ha B.C., Jung J., Kwak B.K. (2015). Susceptibility-weighted imaging for stem cell visualization in a rat photothrombotic cerebral infarction model. Acta Radiol..

[65] Liao N., Wu M., Pan F., Lin J., Li Z., Zhang D., Wang Y., Zheng Y., Peng J., Liu X. (2016). Poly (dopamine) coated superparamagnetic iron oxide nanocluster for noninvasive labeling, tracking, and targeted delivery of adipose tissue-derived stem cells. Sci. Rep..

[66] Napp J., Markus M.A., Heck J.G., Dullin C., Möbius W., Gorpas D., Feldmann C., Alves F. (2018). Therapeutic Fluorescent Hybrid Nanoparticles for Traceable Delivery of Glucocorticoids to Inflammatory Sites. Theranostics.

[67] Ren H., Chen S., Jin Y., Zhang C., Yang X., Ge K., Liang X.-J., Li Z., Zhang J. (2017). A traceable and bone-targeted nanoassembly based on defect-related luminescent mesoporous silica for enhanced osteogenic differentiation. J. Mater. Chem. B.

[68] Hosseinpour S., Cao Y., Liu J., Xu C., Walsh L.J. (2021). Efficient transfection and long-term stability of rno-miRNA-26a-5p for osteogenic differentiation by large pore sized mesoporous silica nanoparticles. J. Mater. Chem. B.

[69] Wu G., Feng C., Quan J., Wang Z., Wei W., Zang S., Kang S., Hui G., Chen X., Wang Q. (2018). In situ controlled release of stromal cell-derived factor-1α and antimiR-138 for on-demand cranial bone regeneration. Carbohydr. Polym..

[70] Ren N., Liang N., Yu X., Wang A., Xie J., Sun C. (2020). Ligand-free upconversion nanoparticles for cell labeling and their effects on stem cell differentiation. Nanotechnology.

[71] Yuan L., Qi X., Qin G., Liu Q., Zhang F., Song Y., Deng J. (2019). Effects of gold nanostructures on differentiation of mesenchymal stem cells. Colloids Surf. B Biointerfaces.

[72] Choi Y.-E., Kwak J.-W., Park J.W. (2010). Nanotechnology for early cancer detection. Sensors.

[73] Chinen A.B., Guan C.M., Ferrer J.R., Barnaby S.N., Merkel T.J., Mirkin C.A. (2015). Nanoparticle Probes for the Detection of Cancer Biomarkers, Cells, and Tissues by Fluorescence. Chem. Rev..

[74] Li H., Mu Y., Qian S., Lu J., Wan Y., Fu G., Liu S. (2015). Synthesis of fluorescent dye-doped silica nanoparticles for target-cell-specific delivery and intracellular microRNA imaging. Analyst.

[75] Chen M.-Y., Chen Z.-Z., Wang W., Zhu L., Tang H.-W., Pang D.-W. (2014). Preparation of RuBpy-doped Silica Fluorescent Nanoprobes and Their Applications to the Recognition of Liver Cancer Cells. Chin. J. Anal. Chem..

[76] Gao J., Zhang Q., Xu J., Guo L., Li X. (2013). Clinical significance of serum miR-21 in breast cancer compared with CA153 and CEA. Chin. J. Cancer Res..

[77] Pedley R.B., Boden J.A., Boden R., Begent R.H., Turner A., Haines A.M., King D.J. (1994). The potential for enhanced tumour localisation by poly(ethylene glycol) modification of anti-CEA antibody. Br. J. Cancer.

[78] Wang R., Degirmenci V., Xin H., Li Y., Wang L., Chen J., Hu X., Zhang D. (2018). PEI-Coated Fe_3_O_4_; Nanoparticles Enable Efficient Delivery of Therapeutic siRNA Targeting REST into Glioblastoma Cells. Int. J. Mol. Sci..

[79] Gao M., Xu H., Bao X., Zhang C., Guan X., Liu H., Lv L., Deng S., Gao D., Wang C. (2016). Oleanolic acid-loaded PLGA-TPGS nanoparticles combined with heparin sodium-loaded PLGA-TPGS nanoparticles for enhancing chemotherapy to liver cancer. Life Sci..

[80] Liu H., Xu H., Zhang C., Gao M., Gao X., Ma C., Lv L., Gao D., Deng S., Wang C. (2016). Emodin-Loaded PLGA-TPGS Nanoparticles Combined with Heparin Sodium-Loaded PLGA-TPGS Nanoparticles to Enhance Chemotherapeutic Efficacy Against Liver Cancer. Pharm. Res..

[81] Zhang B., Sun X., Mei H., Wang Y., Liao Z., Chen J., Zhang Q., Hu Y., Pang Z., Jiang X. (2013). LDLR-mediated peptide-22-conjugated nanoparticles for dual-targeting therapy of brain glioma. Biomaterials.

[82] Yue J., Liu S., Wang R., Hu X., Xie Z., Huang Y., Jing X. (2012). Transferrin-conjugated micelles: Enhanced accumulation and antitumor effect for transferrin-receptor-overexpressing cancer models. Mol. Pharm..

[83] Xiong L., Guo Y., Zhang Y., Cao F. (2016). Highly luminescent and photostable near-infrared fluorescent polymer dots for long-term tumor cell tracking in vivo. J. Mater. Chem. B.

[84] Singh A., Jain S., Senapati S., Verma R.S., Sahoo S.K. (2015). Magnetic Nanoparticles Labeled Mesenchymal Stem Cells: A Pragmatic Solution toward Targeted Cancer Theranostics. Adv. Healthc. Mater..

[85] Shen Y., Zhang J., Hao W., Wang T., Liu J., Xie Y., Xu S., Liu H. (2018). Copolymer micelles function as pH-responsive nanocarriers to enhance the cytotoxicity of a HER2 aptamer in HER2-positive breast cancer cells. Int. J. Nanomed..

[86] Soenen S.J., De Meyer S.F., Dresselaers T., Vande Velde G., Pareyn I.M., Braeckmans K., De Cuyper M., Himmelreich U., Vanhoorelbeke K.I. (2011). MRI assessment of blood outgrowth endothelial cell homing using cationic magnetoliposomes. Biomaterials.

[87] Zhang Y.S., Wang Y., Wang L., Wang Y., Cai X., Zhang C., Wang L.V., Xia Y. (2013). Labeling human mesenchymal stem cells with gold nanocages for in vitro and in vivo tracking by two-photon microscopy and photoacoustic microscopy. Theranostics.

[88] Zaw Thin M., Allan H., Bofinger R., Kostelec T.D., Guillaume S., Connell J.J., Patrick P.S., Hailes H.C., Tabor A.B., Lythgoe M.F. (2020). Multi-modal imaging probe for assessing the efficiency of stem cell delivery to orthotopic breast tumours. Nanoscale.

[89] Li W.-J., Wang Y., Liu Y., Wu T., Cai W.-L., Shuai X.-T., Hong G.-B. (2018). Preliminary Study of MR and Fluorescence Dual-mode Imaging: Combined Macrophage-Targeted and Superparamagnetic Polymeric Micelles. Int. J. Med. Sci..

[90] Foroozandeh P., Aziz A.A. (2018). Insight into Cellular Uptake and Intracellular Trafficking of Nanoparticles. Nanoscale Res. Lett..

[91] Ma X.-H., Wang S., Liu S.-Y., Chen K., Wu Z.-Y., Li D.-F., Mi Y.-T., Hu L.-B., Chen Z.-W., Zhao X.-M. (2019). Development and in vitro study of a bi-specific magnetic resonance imaging molecular probe for hepatocellular carcinoma. World J. Gastroenterol..

[92] Zhang W., Qiao L., Wang X., Senthilkumar R., Wang F., Chen B. (2015). Inducing cell cycle arrest and apoptosis by dimercaptosuccinic acid modified Fe_3_O_4_ magnetic nanoparticles combined with nontoxic concentration of bortezomib and gambogic acid in RPMI-8226 cells. Int. J. Nanomed..

[93] Al Faraj A., Shaik A.S., Al Sayed B., Halwani R., Al Jammaz I. (2016). Specific targeting and noninvasive imaging of breast cancer stem cells using single-walled carbon nanotubes as novel multimodality nanoprobes. Nanomedicine.

[94] Han Y., An Y., Jia G., Wang X., He C., Ding Y., Tang Q. (2018). Facile assembly of upconversion nanoparticle-based micelles for active targeted dual-mode imaging in pancreatic cancer. J. Nanobiotechnol..

[95] Dumoga S., Rai Y., Bhatt A.N., Tiwari A.K., Singh S., Mishra A.K., Kakkar D. (2017). Block Copolymer Based Nanoparticles for Theranostic Intervention of Cervical Cancer: Synthesis, Pharmacokinetics, and in Vitro/in Vivo Evaluation in HeLa Xenograft Models. ACS Appl. Mater. Interfaces.

[96] Jayapaul J., Arns S., Bunker M., Weiler M., Rutherford S., Comba P., Kiessling F. (2016). In vivo evaluation of riboflavin receptor targeted fluorescent USPIO in mice with prostate cancer xenografts. Nano Res..

[97] Pan L., He M., Ma J., Tang W., Gao G., He R., Su H., Cui D. (2013). Phase and size controllable synthesis of NaYbF4 nanocrystals in oleic acid/ionic liquid two-phase system for targeted fluorescent imaging of gastric cancer. Theranostics.

[98] Barar J., Kafil V., Majd M.H., Barzegari A., Khani S., Johari-Ahar M., Asgari D., Coukos G., Cokous G., Omidi Y. (2015). Multifunctional mitoxantrone-conjugated magnetic nanosystem for targeted therapy of folate receptor-overexpressing malignant cells. J. Nanobiotechnol..

[99] Jayapaul J., Hodenius M., Arns S., Lederle W., Lammers T., Comba P., Kiessling F., Gaetjens J. (2011). FMN-coated fluorescent iron oxide nanoparticles for RCP-mediated targeting and labeling of metabolically active cancer and endothelial cells. Biomaterials.

[100] Chang J.-Y., Wang G.-Q., Cheng C.-Y., Lin W.-X., Hsu J.-C. (2012). Strategies for photoluminescence enhancement of AgInS2 quantum dots and their application as bioimaging probes. J. Mater. Chem..

[101] Narmani A., Rezvani M., Farhood B., Darkhor P., Mohammadnejad J., Amini B., Refahi S., Abdi Goushbolagh N. (2019). Folic acid functionalized nanoparticles as pharmaceutical carriers in drug delivery systems. Drug Dev. Res..

[102] Darguzyte M., Drude N., Lammers T., Kiessling F. (2020). Riboflavin-Targeted Drug Delivery. Cancers.

[103] Jiang D., Gao X., Kang T., Feng X., Yao J., Yang M., Jing Y., Zhu Q., Feng J., Chen J. (2016). Actively targeting D-α-tocopheryl polyethylene glycol 1000 succinate-poly(lactic acid) nanoparticles as vesicles for chemo-photodynamic combination therapy of doxorubicin-resistant breast cancer. Nanoscale.

[104] Morita Y., Sakurai R., Wakimoto T., Kobayashi K., Xu B., Toku Y., Song G., Luo Q., Ju Y. (2019). tLyP-1-conjugated core-shell nanoparticles, Fe3O4NPs@mSiO2, for tumor-targeted drug delivery. Appl. Surf. Sci..

[105] Li H., Wang P., Deng Y., Zeng M., Tang Y., Zhu W.-H., Cheng Y. (2017). Combination of active targeting, enzyme-triggered release and fluorescent dye into gold nanoclusters for endomicroscopy-guided photothermal/photodynamic therapy to pancreatic ductal adenocarcinoma. Biomaterials.

[106] Yoon S., Rossi J.J. (2018). Aptamers: Uptake mechanisms and intracellular applications. Adv. Drug Deliv. Rev..

[107] Figueiredo P., Sipponen M.H., Lintinen K., Correia A., Kiriazis A., Yli-Kauhaluoma J., Österberg M., George A., Hirvonen J., Kostiainen M.A. (2019). Preparation and Characterization of Dentin Phosphophoryn-Derived Peptide-Functionalized Lignin Nanoparticles for Enhanced Cellular Uptake. Small.

[108] Hansen L., Hansen A.B., Mathiasen A.B., Ng M., Bhakoo K., Ekblond A., Kastrup J., Friis T. (2014). Ultrastructural characterization of mesenchymal stromal cells labeled with ultrasmall superparamagnetic iron-oxide nanoparticles for clinical tracking studies. Scand. J. Clin. Lab. Investig..

[109] Tong H.-I., Kang W., Shi Y., Zhou G., Lu Y. (2016). Physiological function and inflamed-brain migration of mouse monocyte-derived macrophages following cellular uptake of superparamagnetic iron oxide nanoparticles-Implication of macrophage-based drug delivery into the central nervous system. Int. J. Pharm..

[110] Fidler F., Steinke M., Kraupner A., Gruttner C., Hiller K.-H., Briel A., Westphal F., Walles H., Jakob P.M. (2015). Stem Cell Vitality Assessment Using Magnetic Particle Spectroscopy. IEEE Trans. Magn..

[111] Xu D., Wu F., Chen Y., Wei L., Yuan W. (2013). pH-sensitive degradable nanoparticles for highly efficient intracellular delivery of exogenous protein. Int. J. Nanomed..

[112] Saito S., Tsugeno M., Koto D., Mori Y., Yoshioka Y., Nohara S., Murase K. (2012). Impact of surface coating and particle size on the uptake of small and ultrasmall superparamagnetic iron oxide nanoparticles by macrophages. Int. J. Nanomed..

[113] Nejadnik H., Henning T.D., Castaneda R.T., Boddington S., Taubert S., Jha P., Tavri S., Golovko D., Ackerman L., Meier R. (2012). Somatic differentiation and MR imaging of magnetically labeled human embryonic stem cells. Cell Transplant..

[114] Mo R., Yang J., Wu E.X., Lin S. (2011). Instant magnetic labeling of tumor cells by ultrasound in vitro. J. Magn. Magn. Mater..

[115] Menon L.G., Pratt J., Yang H.W., Black P.M., Sorensen G.A., Carroll R.S. (2012). Imaging of human mesenchymal stromal cells: Homing to human brain tumors. J. Neurooncol..

[116] Cai Q.-Y., Lee H., Kim E.-J., Moon H., Chang K., Rho J., Hong K.S. (2012). Magnetic resonance imaging of superparamagnetic iron oxide-labeled macrophage infiltrates in acute-phase renal ischemia-reperfusion mouse model. Nanomed. Nanotechnol. Biol. Med..

[117] Tong M., Xiong F., Shi Y., Luo S., Liu Z., Wu Z., Wang Z. (2013). In vitro study of SPIO-labeled human pancreatic cancer cell line BxPC-3. Contrast Media Mol. Imaging.

[118] Meir R., Shamalov K., Betzer O., Motiei M., Horovitz-Fried M., Yehuda R., Popovtzer A., Popovtzer R., Cohen C.J. (2015). Nanomedicine for Cancer Immunotherapy: Tracking Cancer-Specific T-Cells in Vivo with Gold Nanoparticles and CT Imaging. ACS Nano.

[119] Meir R., Betzer O., Motiei M., Kronfeld N., Brodie C., Popovtzer R. (2017). Design principles for noninvasive, longitudinal and quantitative cell tracking with nanoparticle-based CT imaging. Nanomed. Nanotechnol. Biol. Med..

[120] Datz S., Illes B., Gößl D., Schirnding C.V., Engelke H., Bein T. (2018). Biocompatible crosslinked β-cyclodextrin nanoparticles as multifunctional carriers for cellular delivery. Nanoscale.

[121] Kröger A.P.P., Komil M.I., Hamelmann N.M., Juan A., Stenzel M.H., Paulusse J.M.J. (2019). Glucose Single-Chain Polymer Nanoparticles for Cellular Targeting. ACS Macro Lett..

[122] Faidah M., Noorwali A., Atta H., Ahmed N., Habib H., Damiati L., Filimban N., Al-Qriqri M., Mahfouz S., Khabaz M.N. (2017). Mesenchymal stem cell therapy of hepatocellular carcinoma in rats: Detection of cell homing and tumor mass by magnetic resonance imaging using iron oxide nanoparticles. Adv. Clin. Exp. Med..

[123] Ning X., Bao H., Liu X., Fu H., Wang W., Huang J., Zhang Z. (2019). Long-term in vivo CT tracking of mesenchymal stem cells labeled with Au@BSA@PLL nanotracers. Nanoscale.

[124] Wang X., Wei F., Liu A., Wang L., Wang J.-C., Ren L., Liu W., Tu Q., Li L., Wang J. (2012). Cancer stem cell labeling using poly(L-lysine)-modified iron oxide nanoparticles. Biomaterials.

[125] Jiang J., Chen Y., Zhu Y., Yao X., Qi J. (2011). Efficient in vitro labeling of human prostate cancer cells with superparamagnetic iron oxide nanoparticles. Cancer Biother. Radiopharm..

[126] Wang S., Fang J., Zhang T., Wang B., Chen J., Li X., Zhang S., Zhang W. (2011). Magnetic resonance imaging targeting of intracranial glioma xenografts by Resovist-labeled endothelial progenitor cells. J. Neurooncol..

[127] Shelat R., Bhatt L.K., Khanna A., Chandra S. (2019). A comprehensive toxicity evaluation of novel amino acid-modified magnetic ferrofluids for magnetic resonance imaging. Amino Acids.

[128] Han Z., Liu S., Pei Y., Ding Z., Li Y., Wang X., Zhan D., Xia S., Driedonks T., Witwer K.W. (2021). Highly efficient magnetic labelling allows MRI tracking of the homing of stem cell-derived extracellular vesicles following systemic delivery. J. Extracell. Vesicles.

[129] Yu L., Bridgers A., Polli J., Vickers A., Long S., Roy A., Winnike R., Coffin M. (1999). Vitamin E-TPGS increases absorption flux of an HIV protease inhibitor by enhancing its solubility and permeability. Pharm. Res..

[130] Burke B.P., Cawthorne C., Archibald S.J. (2017). Multimodal nanoparticle imaging agents: Design and applications. Philos. Transact. A Math. Phys. Eng. Sci..

[131] Sun M., Sun B., Liu Y., Shen Q.-D., Jiang S. (2016). Dual-Color Fluorescence Imaging of Magnetic Nanoparticles in Live Cancer Cells Using Conjugated Polymer Probes. Sci. Rep..

[132] Wu Y., Tang W., Wang P., Liu C., Yuan Y., Qian J. (2015). Cytotoxicity and Cellular Uptake of Amorphous Silica Nanoparticles in Human Cancer Cells. Part. Part. Syst. Charact..

[133] Shahabi S., Treccani L., Dringen R., Rezwan K. (2015). Dual fluorophore doped silica nanoparticles for cellular localization studies in multiple stained cells. Acta Biomater..

[134] Efeoglu E., Keating M., McIntyre J., Casey A., Byrne H.J. (2015). Determination of nanoparticle localisation within subcellular organelles in vitro using Raman spectroscopy. Anal. Methods.

[135] Mumin A.M., Barrett J.W., Dekaban G.A., Zhang J. (2011). Dendritic cell internalization of foam-structured fluorescent mesoporous silica nanoparticles. J. Colloid Interface Sci..

[136] Tang W., Yuan Y., Liu C., Wu Y., Lu X., Qian J. (2014). Differential cytotoxicity and particle action of hydroxyapatite nanoparticles in human cancer cells. Nanomedicine.

[137] Roy D., Mukhuty A., Fouzder C., Bar N., Chowdhury S., Kundu R., Chowdhury P. (2021). Multi-emissive biocompatible silicon quantum dots: Synthesis, characterization, intracellular imaging and improvement of two fold drug efficacy. Dyes Pigment..

[138] Lindemann A., Lüdtke-Buzug K., Fräderich B.M., Gräfe K., Pries R., Wollenberg B. (2014). Biological impact of superparamagnetic iron oxide nanoparticles for magnetic particle imaging of head and neck cancer cells. Int. J. Nanomed..

[139] McCormick S.C., Stillman N., Hockley M., Perriman A.W., Hauert S. (2021). Measuring Nanoparticle Penetration Through Bio-Mimetic Gels. Int. J. Nanomed..

[140] Lee S.H., Park D.J., Yun W.S., Park J.-E., Choi J.S., Key J., Seo Y.J. (2020). Endocytic trafficking of polymeric clustered superparamagnetic iron oxide nanoparticles in mesenchymal stem cells. J. Control. Release.

[141] Smyth P., Gibson T.J., Irvine G., Black G., Lavery D., Semsarilar M., Scott C.J., Themistou E. (2020). pH-Responsive benzaldehyde-functionalized PEG-based polymeric nanoparticles for drug delivery: Effect of preparation method on morphology, dye encapsulation and attachment. Eur. Polym. J..

[142] Luo Y., Liu F., Li E., Fang Y., Zhao G., Dai X., Li J., Wang B., Xu M., Liao B. (2020). FRET-based fluorescent nanoprobe platform for sorting of active microorganisms by functional properties. Biosens. Bioelectron..

[143] Qiu K., Du Y., Liu J., Guan J.-L., Chao H., Diao J. (2020). Super-resolution observation of lysosomal dynamics with fluorescent gold nanoparticles. Theranostics.

[144] Mortimer G.M., Jack K.S., Musumeci A.W., Martin D.J., Minchin R.F. (2016). Stable non-covalent labeling of layered silicate nanoparticles for biological imaging. Mater. Sci. Eng. C Mater. Biol. Appl..

[145] Khalid A., Tran P.A., Norello R., Simpson D.A., O’Connor A.J., Tomljenovic-Hanic S. (2016). Intrinsic fluorescence of selenium nanoparticles for cellular imaging applications. Nanoscale.

[146] Chernenko T., Buyukozturk F., Miljkovic M., Carrier R., Diem M., Amiji M. (2013). Label-Free Raman Microspectral Analysis for Comparison of Cellular Uptake and Distribution between Non-Targeted and EGFR-Targeted Biodegradable Polymeric Nanoparticles. Drug Deliv. Transl. Res..

[147] Zane A., McCracken C., Knight D.A., Young T., Lutton A.D., Olesik J.W., Waldman W.J., Dutta P.K. (2015). Uptake of bright fluorophore core-silica shell nanoparticles by biological systems. Int. J. Nanomed..

[148] Lee C.-M., Lee T.K., Kim D.-I., Kim Y.-R., Kim M.-K., Jeong H.-J., Sohn M.-H., Lim S.T. (2014). Optical imaging of absorption and distribution of RITC-SiO_2_ nanoparticles after oral administration. Int. J. Nanomed..

[149] Kotsuchibashi Y., Zhang Y., Ahmed M., Ebara M., Aoyagi T., Narain R. (2013). Fabrication of FITC-doped silica nanoparticles and study of their cellular uptake in the presence of lectins. J. Biomed. Mater. Res. A.

[150] Zuber A., Purdey M., Schartner E., Forbes C., van der Hoek B., Giles D., Abell A., Monro T., Ebendorff-Heidepriem H. (2016). Detection of gold nanoparticles with different sizes using absorption and fluorescence based method. Sens. Actuators B Chem..

[151] Madsen J., Canton I., Warren N.J., Themistou E., Blanazs A., Ustbas B., Tian X., Pearson R., Battaglia G., Lewis A.L. (2013). Nile Blue-based nanosized pH sensors for simultaneous far-red and near-infrared live bioimaging. J. Am. Chem. Soc..

[152] Kasper J., Hermanns M.I., Bantz C., Koshkina O., Lang T., Maskos M., Pohl C., Unger R.E., Kirkpatrick C.J. (2013). Interactions of silica nanoparticles with lung epithelial cells and the association to flotillins. Arch. Toxicol..

[153] Yang C.-Y., Hsiao J.-K., Tai M.-F., Chen S.-T., Cheng H.-Y., Wang J.-L., Liu H.-M. (2011). Direct labeling of hMSC with SPIO: The long-term influence on toxicity, chondrogenic differentiation capacity, and intracellular distribution. Mol. Imaging Biol..

[154] Liu W.-M., Xue Y.-N., He W.-T., Zhuo R.-X., Huang S.-W. (2011). Dendrimer modified magnetic iron oxide nanoparticle/DNA/PEI ternary complexes: A novel strategy for magnetofection. J. Control. Release.

[155] Dabrowska S., Del Fattore A., Karnas E., Frontczak-Baniewicz M., Kozlowska H., Muraca M., Janowski M., Lukomska B. (2018). Imaging of extracellular vesicles derived from human bone marrow mesenchymal stem cells using fluorescent and magnetic labels. Int. J. Nanomed..

[156] Pužar Dominkuš P., Stenovec M., Sitar S., Lasič E., Zorec R., Plemenitaš A., Žagar E., Kreft M., Lenassi M. (2018). PKH26 labeling of extracellular vesicles: Characterization and cellular internalization of contaminating PKH26 nanoparticles. Biochim. Biophys. Acta Biomembr..

[157] Brown K., Thurn T., Xin L., Liu W., Bazak R., Chen S., Lai B., Vogt S., Jacobsen C., Paunesku T. (2018). Intracellular in situ labeling of TiO_2_ nanoparticles for fluorescence microscopy detection. Nano Res..

[158] Saladino G.M., Vogt C., Li Y., Shaker K., Brodin B., Svenda M., Hertz H.M., Toprak M.S. (2021). Optical and X-ray Fluorescent Nanoparticles for Dual Mode Bioimaging. ACS Nano.

[159] Sweeney S.K., Manzar G.S., Zavazava N., Assouline J.G. (2018). Tracking embryonic hematopoietic stem cells to the bone marrow: Nanoparticle options to evaluate transplantation efficiency. Stem Cell Res. Ther..

[160] Lauridsen H., Foldager C.B., Hansen L., Pedersen M. (2018). Non-invasive cell tracking of SPIO labeled cells in an intrinsic regenerative environment: The axolotl limb. Exp. Ther. Med..

[161] Hsu F.-T., Sun R., Hsieh C.-L. (2019). Cellular Magnetic Resonance Imaging with Superparamagnetic Iron Oxide: Methods and Applications in Cancer. SPIN.

[162] Xu H., Cheng L., Wang C., Ma X., Li Y., Liu Z. (2011). Polymer encapsulated upconversion nanoparticle/iron oxide nanocomposites for multimodal imaging and magnetic targeted drug delivery. Biomaterials.

[163] Andreiuk B., Reisch A., Lindecker M., Follain G., Peyriéras N., Goetz J.G., Klymchenko A.S. (2017). Fluorescent Polymer Nanoparticles for Cell Barcoding In Vitro and In Vivo. Small.

[164] Park J.S., Park W., Park S., Larson A.C., Kim D.-H., Park K.-H. (2017). Multimodal Magnetic Nanoclusters for Gene Delivery, Directed Migration, and Tracking of Stem Cells. Adv. Funct. Mater..

[165] Wang L., Xu K., Hou X., Han Y., Liu S., Wiraja C., Yang C., Yang J., Wang M., Dong X. (2017). Fluorescent Poly(glycerol-co-sebacate) Acrylate Nanoparticles for Stem Cell Labeling and Longitudinal Tracking. ACS Appl. Mater. Interfaces.

[166] Saito A., Mekawy M.M., Sumiyoshi A., Riera J.J., Shimizu H., Kawashima R., Tominaga T. (2016). Noninvasive targeting delivery and in vivo magnetic resonance tracking method for live apoptotic cells in cerebral ischemia with functional Fe2O3 magnetic nanoparticles. J. Nanobiotechnol..

[167] Domey J., Bergemann C., Bremer-Streck S., Krumbein I., Reichenbach J.R., Teichgräber U., Hilger I. (2016). Long-term prevalence of NIRF-labeled magnetic nanoparticles for the diagnostic and intraoperative imaging of inflammation. Nanotoxicology.

[168] Kim S.M., Jeong C.H., Woo J.S., Ryu C.H., Lee J.-H., Jeun S.-S. (2016). In vivo near-infrared imaging for the tracking of systemically delivered mesenchymal stem cells: Tropism for brain tumors and biodistribution. Int. J. Nanomed..

[169] Kim J.S., Kim Y.-H., Kim J.H., Kang K.W., Tae E.L., Youn H., Kim D., Kim S.-K., Kwon J.-T., Cho M.-H. (2012). Development and in vivo imaging of a PET/MRI nanoprobe with enhanced NIR fluorescence by dye encapsulation. Nanomedicine.

[170] Chehade M., Srivastava A.K., Bulte J.W.M. (2016). Co-Registration of Bioluminescence Tomography, Computed Tomography, and Magnetic Resonance Imaging for Multimodal In Vivo Stem Cell Tracking. Tomography.

[171] Qi S., Zhang P., Ma M., Yao M., Wu J., Mäkilä E., Salonen J., Ruskoaho H., Xu Y., Santos H.A. (2019). Cellular Internalization-Induced Aggregation of Porous Silicon Nanoparticles for Ultrasound Imaging and Protein-Mediated Protection of Stem Cells. Small.

[172] Chen M., Betzer O., Fan Y., Gao Y., Shen M., Sadan T., Popovtzer R., Shi X. (2020). Multifunctional Dendrimer-Entrapped Gold Nanoparticles for Labeling and Tracking T Cells Via Dual-Modal Computed Tomography and Fluorescence Imaging. Biomacromolecules.

[173] Chen Y.-C., Wen S., Shang S.-A., Cui Y., Luo B., Teng G.-J. (2014). Magnetic resonance and near-infrared imaging using a novel dual-modality nano-probe for dendritic cell tracking in vivo. Cytotherapy.

[174] Schmidtke-Schrezenmeier G., Urban M., Musyanovych A., Mailänder V., Rojewski M., Fekete N., Menard C., Deak E., Tarte K., Rasche V. (2011). Labeling of mesenchymal stromal cells with iron oxide-poly(L-lactide) nanoparticles for magnetic resonance imaging: Uptake, persistence, effects on cellular function and magnetic resonance imaging properties. Cytotherapy.

[175] Ren Z., Wang J., Zou C., Guan Y., Zhang Y.A. (2011). Labeling of cynomolgus monkey bone marrow-derived mesenchymal stem cells for cell tracking by multimodality imaging. Sci. China Life Sci..

[176] Namestnikova D., Gubskiy I., Kholodenko I., Melnikov P., Sukhinich K., Gabashvili A., Vishnevskiy D., Soloveva A., Abakumov M., Vakhrushev I. (2017). Methodological aspects of MRI of transplanted superparamagnetic iron oxide-labeled mesenchymal stem cells in live rat brain. PLoS ONE.

[177] Li W., Chen R., Lv J., Wang H., Liu Y., Peng Y., Qian Z., Fu G., Nie L. (2018). In Vivo Photoacoustic Imaging of Brain Injury and Rehabilitation by High-Efficient Near-Infrared Dye Labeled Mesenchymal Stem Cells with Enhanced Brain Barrier Permeability. Adv. Sci..

[178] Ma T., Zheng J., Zhang T., Xing D. (2018). Ratiometric photoacoustic nanoprobes for monitoring and imaging of hydrogen sulfide in vivo. Nanoscale.

[179] Abbasi A.Z., Prasad P., Cai P., He C., Foltz W.D., Amini M.A., Gordijo C.R., Rauth A.M., Wu X.Y. (2015). Manganese oxide and docetaxel co-loaded fluorescent polymer nanoparticles for dual modal imaging and chemotherapy of breast cancer. J. Control. Release.

[180] Varma N.R.S., Shankar A., Iskander A., Janic B., Borin T.F., Ali M.M., Arbab A.S. (2013). Differential biodistribution of intravenously administered endothelial progenitor and cytotoxic T-cells in rat bearing orthotopic human glioma. BMC Med. Imaging.

[181] Moon S.-H., Yang B.Y., Kim Y.J., Hong M.K., Lee Y.-S., Lee D.S., Chung J.-K., Jeong J.M. (2016). Development of a complementary PET/MR dual-modal imaging probe for targeting prostate-specific membrane antigen (PSMA). Nanomed. Nanotechnol. Biol. Med..

[182] Kim S., Chae M.K., Yim M.S., Jeong I.H., Cho J., Lee C., Ryu E.K. (2013). Hybrid PET/MR imaging of tumors using an oleanolic acid-conjugated nanoparticle. Biomaterials.

[183] Evertsson M., Kjellman P., Cinthio M., Andersson R., Tran T.A., In’t Zandt R., Grafström G., Toftevall H., Fredriksson S., Ingvar C. (2017). Combined Magnetomotive ultrasound, PET/CT, and MR imaging of 68Ga-labelled superparamagnetic iron oxide nanoparticles in rat sentinel lymph nodes in vivo. Sci. Rep..

[184] Madru R., Tran T.A., Axelsson J., Ingvar C., Bibic A., Ståhlberg F., Knutsson L., Strand S.-E. (2013). (68)Ga-labeled superparamagnetic iron oxide nanoparticles (SPIONs) for multi-modality PET/MR/Cherenkov luminescence imaging of sentinel lymph nodes. Am. J. Nucl. Med. Mol. Imaging.

[185] Yang B.Y., Moon S.-H., Seelam S.R., Jeon M.J., Lee Y.-S., Lee D.S., Chung J.-K., Kim Y.I., Jeong J.M. (2015). Development of a multimodal imaging probe by encapsulating iron oxide nanoparticles with functionalized amphiphiles for lymph node imaging. Nanomedicine.

[186] Malinge J., Géraudie B., Savel P., Nataf V., Prignon A., Provost C., Zhang Y., Ou P., Kerrou K., Talbot J.-N. (2017). Liposomes for PET and MR Imaging and for Dual Targeting (Magnetic Field/Glucose Moiety): Synthesis, Properties, and in Vivo Studies. Mol. Pharm..

[187] Jin L., Wang Q., Chen J., Wang Z., Xin H., Zhang D. (2019). Efficient Delivery of Therapeutic siRNA by Fe_3_O_4_ Magnetic Nanoparticles into Oral Cancer Cells. Pharmaceutics.

[188] Wu M., Zhang H., Tie C., Yan C., Deng Z., Wan Q., Liu X., Yan F., Zheng H. (2018). MR imaging tracking of inflammation-activatable engineered neutrophils for targeted therapy of surgically treated glioma. Nat. Commun..

[189] Zhao J., Vykoukal J., Abdelsalam M., Recio-Boiles A., Huang Q., Qiao Y., Singhana B., Wallace M., Avritscher R., Melancon M.P. (2014). Stem cell-mediated delivery of SPIO-loaded gold nanoparticles for the theranosis of liver injury and hepatocellular carcinoma. Nanotechnology.

[190] Thakkar A., Desai P., Chenreddy S., Modi J., Thio A., Khamas W., Ann D., Wang J., Prabhu S. (2018). Novel nano-drug combination therapeutic regimen demonstrates significant efficacy in the transgenic mouse model of pancreatic ductal adenocarcinoma. Am. J. Cancer Res..

[191] Afonso D., Mascarenhas V. (2015). Imaging techniques for the diagnosis of soft tissue tumors. Rep. Med. Imaging.

[192] Ladd L.M., Roth T.D. (2017). Computed Tomography and Magnetic Resonance Imaging of Bone Tumors. Semin. Roentgenol..

[193] Hillman E.M.C., Amoozegar C.B., Wang T., McCaslin A.F.H., Bouchard M.B., Mansfield J., Levenson R.M. (2011). In vivo optical imaging and dynamic contrast methods for biomedical research. Philos. Trans. R. Soc. Math. Phys. Eng. Sci..

[194] Ljungberg M., Pretorius P.H. (2018). SPECT/CT: An update on technological developments and clinical applications. Br. J. Radiol..

[195] Barca C., Griessinger C.M., Faust A., Depke D., Essler M., Windhorst A.D., Devoogdt N., Brindle K.M., Schäfers M., Zinnhardt B. (2021). Expanding Theranostic Radiopharmaceuticals for Tumor Diagnosis and Therapy. Pharmaceuticals.

[196] Mushtaq S., Bibi A., Park J.E., Jeon J. (2021). Recent Progress in Technetium-99m-Labeled Nanoparticles for Molecular Imaging and Cancer Therapy. Nanomaterials.

[197] Coenen H.H., Ermert J. (2021). Expanding PET-applications in life sciences with positron-emitters beyond fluorine-18. Nucl. Med. Biol..

[198] Fernández-Barahona I., Muñoz-Hernando M., Pellico J., Ruiz-Cabello J., Herranz F. (2018). Molecular Imaging with 68Ga Radio-Nanomaterials: Shedding Light on Nanoparticles. Appl. Sci..

[199] Psimadas D., Georgoulias P., Valotassiou V., Loudos G. (2012). Molecular nanomedicine towards cancer: ^111^In-labeled nanoparticles. J. Pharm. Sci..

[200] Chakravarty R., Chakraborty S. (2021). A review of advances in the last decade on targeted cancer therapy using 177Lu: Focusing on 177Lu produced by the direct neutron activation route. Am. J. Nucl. Med. Mol. Imaging.

[201] Lamb J., Holland J.P. (2018). Advanced Methods for Radiolabeling Multimodality Nanomedicines for SPECT/MRI and PET/MRI. J. Nucl. Med..

[202] Gao H., Liu X., Tang W., Niu D., Zhou B., Zhang H., Liu W., Gu B., Zhou X., Zheng Y. (2016). 99mTc-conjugated manganese-based mesoporous silica nanoparticles for SPECT, pH-responsive MRI and anti-cancer drug delivery. Nanoscale.

[203] Xue S., Zhang C., Yang Y., Zhang L., Cheng D., Zhang J., Shi H., Zhang Y. (2015). 99mTc-Labeled Iron Oxide Nanoparticles for Dual-Contrast (T1/T2) Magnetic Resonance and Dual-Modality Imaging of Tumor Angiogenesis. J. Biomed. Nanotechnol..

[204] Li X., Xiong Z., Xu X., Luo Y., Peng C., Shen M., Shi X. (2016). (99m)Tc-Labeled Multifunctional Low-Generation Dendrimer-Entrapped Gold Nanoparticles for Targeted SPECT/CT Dual-Mode Imaging of Tumors. ACS Appl. Mater. Interfaces.

[205] Belderbos S., González-Gómez M.A., Cleeren F., Wouters J., Piñeiro Y., Deroose C.M., Coosemans A., Gsell W., Bormans G., Rivas J. (2020). Simultaneous in vivo PET/MRI using fluorine-18 labeled Fe_3_O_4_@Al(OH)_3_ nanoparticles: Comparison of nanoparticle and nanoparticle-labeled stem cell distribution. EJNMMI Res..

[206] Wang Y., Liu H., Yao D., Li J., Yang S., Zhang C., Chen W., Wang D. (2019). 18F-labeled magnetic nanoparticles for monitoring anti-angiogenic therapeutic effects in breast cancer xenografts. J. Nanobiotechnol..

[207] Crimì F., Valeggia S., Baffoni L., Stramare R., Lacognata C., Spolverato G., Albertoni L., Spimpolo A., Evangelista L., Zucchetta P. (2021). [18F]FDG PET/MRI in rectal cancer. Ann. Nucl. Med..

[208] Umutlu L., Beyer T., Grueneisen J.S., Rischpler C., Quick H.H., Veit-Haibach P., Eiber M., Purz S., Antoch G., Gatidis S. (2019). Whole-Body [18F]-FDG-PET/MRI for Oncology: A Consensus Recommendation. ROFO Fortschr. Geb. Rontgenstr. Nuklearmed..

[209] Hsu F.-T., Wei Z.-H., Hsuan Y.C.-Y., Lin W., Su Y.-C., Liao C.-H., Hsieh C.-L. (2018). MRI tracking of polyethylene glycol-coated superparamagnetic iron oxide-labelled placenta-derived mesenchymal stem cells toward glioblastoma stem-like cells in a mouse model. Artif. Cells Nanomed. Biotechnol..

[210] Qiao Y., Gumin J., MacLellan C.J., Gao F., Bouchard R., Lang F.F., Stafford R.J., Melancon M.P. (2018). Magnetic resonance and photoacoustic imaging of brain tumor mediated by mesenchymal stem cell labeled with multifunctional nanoparticle introduced via carotid artery injection. Nanotechnology.

[211] Duan L., Zuo J., Zhang F., Li B., Xu Z., Zhang H., Yang B., Song W., Jiang J. (2020). Magnetic Targeting of HU-MSCs in the Treatment of Glucocorticoid-Associated Osteonecrosis of the Femoral Head Through Akt/Bcl2/Bad/Caspase-3 Pathway. Int. J. Nanomed..

[212] Papadimitriou N., Thorfve A., Brantsing C., Junevik K., Baranto A., Barreto Henriksson H. (2014). Cell viability and chondrogenic differentiation capability of human mesenchymal stem cells after iron labeling with iron sucrose. Stem Cells Dev..

[213] Kim S.J., Lewis B., Steiner M.-S., Bissa U.V., Dose C., Frank J.A. (2016). Superparamagnetic iron oxide nanoparticles for direct labeling of stem cells and in vivo MRI tracking. Contrast Media Mol. Imaging.

[214] Hachani R., Birchall M.A., Lowdell M.W., Kasparis G., Tung L.D., Manshian B.B., Soenen S.J., Gsell W., Himmelreich U., Gharagouzloo C.A. (2017). Assessing cell-nanoparticle interactions by high content imaging of biocompatible iron oxide nanoparticles as potential contrast agents for magnetic resonance imaging. Sci. Rep..

[215] Aşık E., Aslan T.N., Güray N.T., Volkan M. (2018). Cellular uptake and apoptotic potential of rhenium labeled magnetic protein cages in MDA-MB-231 cells. Environ. Toxicol. Pharmacol..

[216] Salingova B., Simara P., Matula P., Zajickova L., Synek P., Jasek O., Veverkova L., Sedlackova M., Nichtova Z., Koutna I. (2019). The Effect of Uncoated SPIONs on hiPSC-Differentiated Endothelial Cells. Int. J. Mol. Sci..

[217] Ali A.A.A., Shahror R.A., Chen K.-Y. (2020). Efficient Labeling Of Mesenchymal Stem Cells For High Sensitivity Long-Term MRI Monitoring In Live Mice Brains. Int. J. Nanomed..

[218] Zhang L., Xiao S., Kang X., Sun T., Zhou C., Xu Z., Du M., Zhang Y., Wang G., Liu Y. (2021). Metabolic Conversion and Removal of Manganese Ferrite Nanoparticles in RAW264.7 Cells and Induced Alteration of Metal Transporter Gene Expression. Int. J. Nanomed..

[219] Su H., Mou Y., An Y., Han W., Huang X., Xia G., Ni Y., Zhang Y., Ma J., Hu Q. (2013). The migration of synthetic magnetic nanoparticle labeled dendritic cells into lymph nodes with optical imaging. Int. J. Nanomed..

[220] Chang Y.-K., Liu Y.-P., Ho J.H., Hsu S.-C., Lee O.K. (2012). Amine-surface-modified superparamagnetic iron oxide nanoparticles interfere with differentiation of human mesenchymal stem cells. J. Orthop. Res..

[221] Zhu X.-M., Wang Y.-X.J., Leung K.C.-F., Lee S.-F., Zhao F., Wang D.-W., Lai J.M.Y., Wan C., Cheng C.H.K., Ahuja A.T. (2012). Enhanced cellular uptake of aminosilane-coated superparamagnetic iron oxide nanoparticles in mammalian cell lines. Int. J. Nanomed..

[222] Huang C., Neoh K.G., Wang L., Kang E.-T., Shuter B. (2011). Surface functionalization of superparamagnetic nanoparticles for the development of highly efficient magnetic resonance probe for macrophages. Contrast Media Mol. Imaging.

[223] Mou Y., Chen B., Zhang Y., Hou Y., Xie H., Xia G., Tang M., Huang X., Ni Y., Hu Q. (2011). Influence of synthetic superparamagnetic iron oxide on dendritic cells. Int. J. Nanomed..

[224] Al Faraj A., Luciani N., Kolosnjaj-Tabi J., Mattar E., Clement O., Wilhelm C., Gazeau F. (2013). Real-time high-resolution magnetic resonance tracking of macrophage subpopulations in a murine inflammation model: A pilot study with a commercially available cryogenic probe. Contrast Media Mol. Imaging.

[225] Talukdar Y., Rashkow J., Lalwani G., Kanakia S., Sitharaman B. (2014). The effects of graphene nanostructures on mesenchymal stem cells. Biomaterials.

[226] Barbillon G. (2020). Latest Novelties on Plasmonic and Non-Plasmonic Nanomaterials for SERS Sensing. Nanomaterials.

[227] Donahue N.D., Francek E.R., Kiyotake E., Thomas E.E., Yang W., Wang L., Detamore M.S., Wilhelm S. (2020). Assessing nanoparticle colloidal stability with single-particle inductively coupled plasma mass spectrometry (SP-ICP-MS). Anal. Bioanal. Chem..

[228] Herd H., Daum N., Jones A.T., Huwer H., Ghandehari H., Lehr C.-M. (2013). Nanoparticle geometry and surface orientation influence mode of cellular uptake. ACS Nano.

[229] Sousa de Almeida M., Susnik E., Drasler B., Taladriz-Blanco P., Petri-Fink A., Rothen-Rutishauser B. (2021). Understanding nanoparticle endocytosis to improve targeting strategies in nanomedicine. Chem. Soc. Rev..

[230] Zhao J., Stenzel M.H. (2018). Entry of nanoparticles into cells: The importance of nanoparticle properties. Polym. Chem..

[231] Kafshgari M.H., Harding F.J., Voelcker N.H. (2015). Insights into cellular uptake of nanoparticles. Curr. Drug Deliv..

[232] Behzadi S., Serpooshan V., Tao W., Hamaly M.A., Alkawareek M.Y., Dreaden E.C., Brown D., Alkilany A.M., Farokhzad O.C., Mahmoudi M. (2017). Cellular uptake of nanoparticles: Journey inside the cell. Chem. Soc. Rev..

[233] Kinnear C., Moore T.L., Rodriguez-Lorenzo L., Rothen-Rutishauser B., Petri-Fink A. (2017). Form Follows Function: Nanoparticle Shape and Its Implications for Nanomedicine. Chem. Rev..

[234] Moore T.L., Urban D.A., Rodriguez-Lorenzo L., Milosevic A., Crippa F., Spuch-Calvar M., Balog S., Rothen-Rutishauser B., Lattuada M., Petri-Fink A. (2019). Nanoparticle administration method in cell culture alters particle-cell interaction. Sci. Rep..

[235] Zhang Y., Zhang H., Li B., Zhang H., Tan B., Deng Z. (2018). Cell-assembled (Gd-DOTA)i-triphenylphosphonium (TPP) nanoclusters as a T2 contrast agent reveal in vivo fates of stem cell transplants. Nano Res..

[236] Wan D., Chen D., Li K., Qu Y., Sun K., Tao K., Dai K., Ai S. (2016). Gold Nanoparticles as a Potential Cellular Probe for Tracking of Stem Cells in Bone Regeneration Using Dual-Energy Computed Tomography. ACS Appl. Mater. Interfaces.

[237] Liesveld J.L., Sharma N., Aljitawi O.S. (2020). Stem cell homing: From physiology to therapeutics. Stem Cells.

[238] Schlorf T., Meincke M., Kossel E., Glüer C.-C., Jansen O., Mentlein R. (2010). Biological properties of iron oxide nanoparticles for cellular and molecular magnetic resonance imaging. Int. J. Mol. Sci..

[239] Astolfo A., Schültke E., Menk R.H., Kirch R.D., Juurlink B.H.J., Hall C., Harsan L.-A., Stebel M., Barbetta D., Tromba G. (2013). In vivo visualization of gold-loaded cells in mice using x-ray computed tomography. Nanomed. Nanotechnol. Biol. Med..

[240] Reifschneider O., Vennemann A., Buzanich G., Radtke M., Reinholz U., Riesemeier H., Hogeback J., Köppen C., Großgarten M., Sperling M. (2020). Revealing Silver Nanoparticle Uptake by Macrophages Using SR-μXRF and LA-ICP-MS. Chem. Res. Toxicol..

[241] Hu G., Guo M., Xu J., Wu F., Fan J., Huang Q., Yang G., Lv Z., Wang X., Jin Y. (2019). Nanoparticles Targeting Macrophages as Potential Clinical Therapeutic Agents Against Cancer and Inflammation. Front. Immunol..

[242] Siegers G.M., Ribot E.J., Keating A., Foster P.J. (2013). Extensive expansion of primary human gamma delta T cells generates cytotoxic effector memory cells that can be labeled with Feraheme for cellular MRI. Cancer Immunol. Immunother. CII.

[243] Kim M.H., Lee Y.J., Kang J.H. (2016). Stem Cell Monitoring with a Direct or Indirect Labeling Method. Nucl. Med. Mol. Imaging.

[244] Elliott A.D. (2020). Confocal Microscopy: Principles and Modern Practices. Curr. Protoc. Cytom..

[245] Berthold F., Tarkkanen V. (2013). Luminometer development in the last four decades: Recollections of two entrepreneurs. Luminescence.

[246] Stokes Debbie J. (2008). Principles and Practice of Variable Pressure Environmental Scanning Electron Microscopy (VP-ESEM). Chichester.

[247] Manohar S.M., Shah P., Nair A. (2021). Flow cytometry: Principles, applications and recent advances. Bioanalysis.

