# Supplementary material for: Nanotechnology-Assisted Cell Tracking
Source: Nanomaterials (Basel). 2022 Apr 20;12(9):1414. doi: 10.3390/nano12091414 (PMC9103829; doi:10.3390/nano12091414)
Supplement: Supplementary file 1 [file nanomaterials-12-01414-s001.zip › Supplementary Tables S1, S3 and S4.pdf]

nanomaterials

Supplementary Materials

Nanotechnology-Assisted Cell Tracking

Alessia Peserico *, Chiara Di Berardino, Valentina Russo, Giulia Capacchietti, Oriana Di Giacinto, Angelo Canciello, Chiara Camerano Spelta Rapini and Barbara Barboni

Faculty of Bioscience and Technology for Food, Agriculture and Environment, University of Teramo, 64100 Teramo, Italy; cdiberardino@unite.it (C.D.B.); vrusso@unite.it (V.R.); gcapacchietti@unite.it (G.C.); odigiacinto@unite.it (O.D.G.); acanciello@unite.it (A.C.); chiara.cameranospelta@studenti.unite.it (C.C.S.R.); bbarboni@unite.it (B.B.)

* Correspondence: apeserico@unite.it

Supplementary Table S1. Clinical Trials studies investigating the use of magnetic NP for cell tracking**.** Interventions distinguish the suitability of magnetic NP application for (1) setting up new procedures [procedures], (2) therapeutic purposes [drug], (3) diagnosis [diagnostic test], (4) the development of new imaging tools [devices].

Title	Conditions	Interventions
Ferumoxytol - Iron Oxide Nanoparticle Magnetic Resonance Dynamic Contrast Enhanced MRI	Head and Neck Cancer	Procedure: MRI<br>Drug: Ferumoxytol
MRI/US Fusion Imaging and Biopsy in Combination With Nanoparticle Directed Focal Therapy for Ablation of Prostate Tissue	Neoplasm of the prostate	Device: AuroShell particle infusion
An Extension Study MRI/US Fusion Imaging and Biopsy in Combination With Nanoparticle Directed Focal Therapy for Ablation of Prostate Tissue	Neoplasm of the prostate	Device: AuroShell particle infusion
A Validation Study of MR Lymphangiography Using SPIO, a New Lymphotropic Superparamagnetic Nanoparticle Contrast	Bladder Cancer<br>Genitourinary Cancer<br>Prostate Cancer	Procedure: MRI lymphangiography<br>Drug: Ferumoxtran-10 (USPIO)
Clinical and Technical Feasibility of a Ultrasuperparamagnetic Nanoparticle Iron Oxide (USPIO)-Enhanced Magnetic Resonance Lymph Node Imaging	Cancer of Lymph Node	Procedure: MRI<br>Drug: Feraheme
IRon Nanoparticle Enhanced MRI in the Assessment of Myocardial infarctioN	Myocardial Infarction<br>Inflammation	Device: Ferumoxytol enhanced MRI

Preoperative Detection of Lymph Node Metastases in Pancreatic and Periampullary Carcinoma Using USPIO MRI	Pancreatic Cancer<br>Periampullary Cancer	Diagnostic Test: USPIO-enhanced MRI
The Use of Nanoparticles to Guide the Surgical Treatment of Prostate Cancer	Prostate cancer	Diagnostic Test: PET/MRI<br>Drug: (64Cu)-labeled PSMA-targeting particle tracer, or 64Cu-NOTA-PSMAi-PEG-Cy5.5-C' dots
Phase II NCT (Neoadjuvant Chemotherapy) w/ Weekly Abraxane in Combination With Carboplatin & Bevacizumab in Breast Cancer	Breast cancer	Procedure:Surgery<br>Drug:bevacizumab,carboplatin, nab-paclitaxel, adjuvant chemotherapy
NBTXR3 Crystalline Nanoparticles and Radiation Therapy in Treating Randomized Patients in Two Arms With Soft Tissue Sarcoma of the Extremity and Trunk Wall	Adult Soft Tissue Sarcoma	Device: NBTXR3<br>Device: Radiation therapy
S1505: Combination Chemotherapy or Gemcitabine Hydrochloride and Paclitaxel Albumin-Stabilized Nanoparticle Formulation Before Surgery in Treating Patients With Pancreatic Cancer That Can Be Removed by Surgery	Pancreatic Adenocarcinoma<br>•Resectable Pancreatic Carcinoma	Procedure: Pancreatectomy<br>Drug: Fluorouracil, Gemcitabine Hydrochloride, Irinotecan Hydrochloride, Oxaliplatin, Paclitaxel Albumin-Stabilized Nanoparticle Formulation

Supplementary Table S3. General operational principles of the technologies used for in vitro cell tracking.

In vitro tracking				
	**Confocal microscope**	**Luminometer**	**SEM and TEM**	**Flow Cytometry**
**Operational principle**	The confocal microscope uses fluorescence optics and a laser as point of illumination. Instead of illuminating the whole sample, laser light is focused onto a defined spot at a specific depth within the sample. This leads to the emission of fluorescent light at exactly this point. A pinhole inside the optical pathway cuts off signals that are out of focus, thus allowing only the fluorescence signals from the illuminated spot to enter the light detector. The detector is a photomultiplier tube (PMT) n which photons are converted to electrons, with the resulting current proportional to the amount of fluorescence. By scanning the specimen, images of one single optical plane are created. 3D objects can be visualized by scanning several optical planes and stacking them using a suitable microscopy deconvolution software (z-stack). Confocal microscopy quantifies components within cellular compartments showing how they are distributed.	A luminometer is an instrument that measures weak emissions of luminescence coming from a sample. Luminescence is the generation of electromagnetic radiation as ultraviolet, visible, or infrared light by the release of energy from a chemical reaction. Unlike fluorescence, where a molecule emits light upon excitation by an external light source, luminescence is generated by a chemical or biological reaction, for example an enzyme and its substrate. The resulting light can be detected by a PMT, in which photons are converted to electrons, with the resulting current proportional to the amount of light. Measurement of the signal is expressed as relative light units (RLU).	SEM and TEM operates on the same basic principles as the light microscope but uses electrons instead of light.<br>SEM is a kind of electron microscope that uses a fine beam of focused electrons to scan a sample's surface. The microscope records information about the interaction between the electrons and the sample, creating a magnified image. SEM has the potential to magnify an image up to 2 million times.<br>TEM can stand for Transmission Electron Microscopy or Transmission Electron Microscope (TEM). TEM is a type of electron microscope that uses a broad beam of electrons to create an image of a sample's internal structure. A beam of electrons is transmitted through a sample, creating an image that details a sample's morphology, composition, and crystal structure.	Flow cytometry is a technique which enables rapid analysis of statistically significant number of cells at single cell level. The main principle of this technique is based on scattering of light and emission of fluorescence which occur when a laser beam hits the cells moving in a directed fluid stream. It is used to detect, count, and cell sorting. Flow cytometry quantify cellular component, on a whole cell level without specifying where the component is exactly located inside the cell.

			TEMs have an incredible magnification potential of 10-50 million time	
**Light source**	Laser	Any	electrons	Laser
**Detected signal**	Fluorescence	ultraviolet, visible or infrared light	SEM: backscattered electrons and secondary electrons<br><br>TEM: transmitted electrons	Fluorescence
**Optimal spatial resolution**	180 nm laterally and 500 nm axially	Not applicable	SEM: ~0.5 nm<br><br>TEM: <50 pm	Not applicable
**Reference**	[244]	[245]	[246]	[247]

Supplementary Table S4. General operational principles of the technologies used for in vivo cell tracking.

In vivo tracking						
	**MRI**	**Tomography imaging**			**In vivo optical imaging**	**Nuclear imaging**
		**CT**	**PA**	**XFT**	**BLI or FLI**	**SPECT or PET**
**Operational principle**	MRI involves imaging protons in vivo. Protons emit a signal when a radio frequency pulse is applied in a magnetic field. MRI employ powerful magnets which produce a strong magnetic field that forces protons in the body to align with that field. When a radiofrequency current is then pulsed through the patient, the protons are stimulated, and spin out of equilibrium, straining against the pull of the magnetic field. When the radiofrequency field is turned off, the MRI sensors are able to detect the energy released as the protons realign with the magnetic field and form an image of these signals. Element such as Gadolinium may be given to a patient intravenously before or during the MRI as contrite agents to increase the speed at which protons realign with the magnetic field. The faster the protons realign, the brighter the image.	CT is based on the fundamental principle that the density of the tissue passed by the x-ray beam can be measured from the calculation of the attenuation coefficient.<br>Unlike a conventional x-ray, which uses a fixed x-ray tube, CT uses a narrow beam of x-rays which is aimed at a patient and quickly rotated around the body, producing signals that are processed by the machine's computer to generate cross-sectional images of the body.<br>Contrast agents as iodinate compounds can be used to improve resolution as contain substances that are better at stopping x-rays	PA uses pulsed laser light to irradiate tissues and, as a result, pressure waves are produced due to the increased temperature and volume. A high-frequency ultrasound transducer monitors these pressure waves, and a 3D reconstruction is performed.	This method uses x-ray radiation to trigger XRF in an object, either from naturally occurring elements or from some inorganic NP. Fluorescent X-rays are electromagnetic waves that are created when irradiated X-rays force inner-shell electrons of the constituent atoms to an outer shell and outer shell electrons promptly move to inner shells to fill the vacancies.	In vivo optical imaging relies on the acquisition of photographic image of the body under white light which allow to quantify bioluminescent (BLI; bioluminescent imaging) or fluorescent (FLI; fluorescent imaging) signal, which is overlaid on the image. The bioluminescent or fluorescent signal is expressed in photons per second and displayed as an intensity map.	Nuclear imaging is used to have metabolic and functional information of a tissue or organ. Single photon emission computed tomography (SPECT) and positron emission tomography (PET) are nuclear medicine imaging techniques. These approaches require the use of radioactive substance, known as radionuclide to assist in the exam. Its spatial and temporal distribution in the body reflects a particular body function or metabolism. The main difference between SPECT and PET scans is the type of radiotracers used. While SPECT scans measure gamma rays, the decay of the radiotracers used with PET scans produce small particles called positrons. The gamma or the positron camera records the energy emissions from

		and, thus, are more visible on an x-ray image. Micro-CT is on a much smaller scale with greatly increased resolution.				the radiotracer absorbed by the body and converts it into an image.
**Signal stimulation**	radio waves	X-rays	LED laser	x-rays	BLI: gene expressing a luminescent element<br>FLI: laser	radionuclides
**Detected signal**	protons	attenuation coefficient	Sound waves	Electromagnetic waves	BLI: Luminescence<br>FLI: Fluorescence	SPECT: Gamma rays<br>PET: Positrons
**Optimal target tissue**	soft	hard	soft	soft	soft	Soft and hard
**Reference**	[89]	[209]	[210]	[211]	[170]	[212]
